# Validation of EUCAST 2026 breakpoints for *Brucella* species using routine laboratory methods: A comprehensive French National Reference Centre study

**DOI:** 10.1371/journal.pntd.0014817

**Published:** 2026-10-27

**Authors:** Marie Gaillard, Théo Pastre, Chloé Magnan, Albert Sotto, David O’Callaghan, Anne Keriel, Jean-Philippe Lavigne

**Affiliations:** 1 Virulence Bactérienne et Infections Chroniques, INSERM U1047, Univ Montpellier, Service de Microbiologie et Hygiène Hospitalière, Centre National de Référence des Brucelloses, CHU Nîmes, Nîmes, France; 2 Virulence Bactérienne et Infections Chroniques, INSERM U1047, Univ Montpellier, Service des Maladies Infectieuses et Tropicales, Centre National de Référence des Brucelloses, CHU Nîmes, Nîmes, France; Colorado State University, UNITED STATES OF AMERICA

## Abstract

Brucellosis, caused by various species of the bacterial genus *Brucella*, represents a significant global health burden in many parts of the world. The European Committee on Antimicrobial Susceptibility Testing (EUCAST) recently established standardized clinical breakpoints (version 16.1, 2026) for *Brucella* to support global surveillance and evidence-based treatment. This study validates EUCAST 2026 breakpoints using routine laboratory methods in a large national reference collection. We retrospectively analyzed 234 *Brucella* isolates from the French National Reference Centre between January 2017 and June 2026, using MALDI-TOF mass spectrometry and multiplex PCR for species identification, and E-test strips and disk diffusion for antimicrobial susceptibility testing (AST) according to EUCAST guidelines. *Brucella melitensis* predominated (n = 210, 89.7%), with smaller numbers of *B. suis*, *B. abortus*, and *B. amazoniensis*. Universal susceptibility was observed across all 234 isolates for eight tested antibiotics. Notably, 49.6% of isolates displayed rifampicin minimum inhibitory concentrations (MICs) within two doubling dilutions of the EUCAST epidemiological cut-off (ECOFF) value, highlighting the need for continued surveillance of rifampicin susceptibility in this genus. A critical methodological finding emerged regarding streptomycin assessment. E-test strips indicated elevated streptomycin MICs in a substantial proportion of isolates, with results consistent across different lots. However, disk diffusion performed on identical isolates demonstrated universal susceptibility for all 234 isolates, with inhibition zone diameters ranging from 21–35 mm. This study validates that EUCAST 2026 breakpoints are clinically appropriate and epidemiologically sound when applied using appropriate routine laboratory methods. Disk diffusion should be considered the reference method for streptomycin susceptibility determination in *Brucella* species. These findings provide essential practical guidance for clinical microbiology laboratories, demonstrate that France remains free from antimicrobial resistance, and support standardized AST testing for optimized management of human brucellosis in both endemic and non-endemic regions worldwide.

## Author summary

Brucellosis is a serious infectious disease that affects livestock and spreads to humans through contact with infected animals or contaminated animal products. The disease is particularly common in Africa and the Middle East but can occur anywhere due to travel and trade. To treat brucellosis effectively, it is needed to know which antibiotics work best against the bacteria causing the disease. In this study, we tested 234 bacterial samples collected in France over eight years to determine which antibiotics were most effective. We found that all bacteria tested remained susceptible to our standard antibiotics. However, we discovered an important problem: one testing method commonly used in laboratories gave misleading results for one antibiotic called streptomycin. When we used a different, more reliable testing method, we confirmed that streptomycin still works well against these bacteria. Our findings help laboratories choose the best testing methods and confirm that the international treatment guidelines being used are working well. Additionally, we found that half of the bacteria had antibiotic levels just below the danger zone, suggesting we need to continue monitoring for potential resistance in the future.

## Introduction

Brucellosis, caused by various species of the bacterial genus *Brucella*, is one of the most widespread zoonotic infections worldwide [1–3]. Recent estimates suggest that the global incidence may range from 2.43 to 7.45 million cases annually [4]. The disease is endemic in several regions, particularly the Mediterranean Basin, the Middle East, Central Asia, Africa, and Central and South America, where it continues to pose a major public health challenge [5,6]. In contrast, brucellosis is rare in most European countries. In 2023, 259 confirmed cases were reported across the European Union and European Economic Area, with a notification rate of 0.06 per 100,000 population, and Greece, Portugal, Italy, Slovakia, and Spain reported the highest numbers of autochthonous human cases. Notably, a 14.1% increase in the notification rate has been observed since 2023, according to the European Food Safety Authority (EFSA) [7]. In France, fewer than 50 cases are reported annually, with the vast majority being imported, predominantly from North Africa, particularly Algeria.

Human infection typically occurs through consumption of unpasteurized dairy products or undercooked meat, direct contact with infected animals, or inhalation of contaminated aerosols, especially in occupational settings [5,6]. Clinical manifestations are highly variable and often non-specific; however, osteoarticular involvement remains the most frequent focal complication [5,6]. The risk of relapse, estimated at 5–15% [8], emphasizes the critical importance of adequate and sustained antimicrobial therapy.

Current treatment guidelines from the World Health Organization (WHO) and the U.S. Centers for Disease Control and Prevention (CDC) recommend dual or triple antimicrobial regimens. Doxycycline and rifampicin form the cornerstone of therapy, while alternatives— including streptomycin, gentamicin, trimethoprim-sulfamethoxazole, ceftriaxone, or fluoroquinolones—are reserved for complicated cases or when contraindications exist [1,2,5,9]. Although *Brucella* spp. are generally considered susceptible to these agents, emerging reports of antimicrobial resistance—particularly to rifampicin and trimethoprim-sulfamethoxazole—from diverse geographic regions highlight the pressing need for standardized antimicrobial susceptibility testing (AST) protocols and clearly defined clinical breakpoints [10–13]. Reduced susceptibility to rifampicin is of particular clinical concern, because rifampicin is a backbone agent of every first-line regimen and because relapse in brucellosis is driven principally by inadequate intracellular killing. Even modest upward shifts in rifampicin MIC may therefore translate into slower clinical response, treatment failure and relapse rather than into overt in vitro resistance. The recent establishment of EUCAST clinical breakpoints for *Brucella* (version 16.1, 2026) [14] represents a significant advance in addressing this long-standing gap in laboratory standardization.

Importantly, brucellosis cannot be controlled by human medicine alone. Because human infection is almost entirely a spill-over from infected small ruminants, cattle and swine, any durable reduction of the human burden depends on a One Health strategy that deliberately couples the human, animal and environmental health sectors: coordinated livestock vaccination and test-and-slaughter programmes, milk pasteurization, regulation of animal movement and trade, protection of occupationally exposed workers, and shared human-veterinary laboratory and surveillance networks. This integrated approach is now recognized as the decisive lever for the control of neglected zoonotic diseases more broadly [15]. Standardized antimicrobial susceptibility testing is an integral component of that strategy, since harmonized and comparable laboratory data are the only means of detecting, comparing and responding to emerging resistance across sectors, regions and countries.

In this study, we analyzed 234 *Brucella* isolates collected between January 2017 and June 2026 by the French National Reference Centre for Brucellosis. Our objectives were to determine the minimum inhibitory concentrations (MICs) of nine clinically relevant antibiotics, validate the newly established EUCAST 2026 breakpoints across various *Brucella* species, assess epidemiological cut-off (ECOFF) value distributions to inform surveillance for emerging resistance, and evaluate the current antimicrobial susceptibility profile of *Brucella* isolates in France.

## Methods

### Ethics

This study was conducted in accordance with the principles of the Declaration of Helsinki. Ethical approval was obtained from the Institutional Review Board (IRB) of Nîmes University Hospital (approval number 2024.03.05). Individual informed consent was not required for this study. In accordance with Article L1211-2 of the French Public Health Code, which governs the secondary use for research of biological samples obtained in the course of care, patients are verbally informed of such use and may object to it, rather than being asked to give consent. Because the National Reference Centre has no contact with patients, this information is delivered by the referring laboratory, and any objection is recorded in a dedicated field of the standardized analysis request form that accompanies every sample sent to the Centre; samples for which an objection had been recorded were excluded from the study. All isolates and associated clinical data were anonymized prior to analysis in accordance with French data protection regulations (Commission Nationale de l’Informatique et des Libertés, CNIL). Data were accessed for research purposes on 9 June 2026.

### Study design and clinical data

We conducted a retrospective observational study of all consecutive *Brucella* isolates submitted to the French National Reference Centre for Brucellosis (Nîmes University Hospital, France) between 1^st^ January 2017 and 15^th^ June 2026. Information regarding the referring laboratories and specimen sources was recorded. Clinical and epidemiological data—including patient age, occupation, history of travel or residence in endemic areas, clinical presentation, and documentation of prior relapse—were collected using a standardized questionnaire accompanying each isolate or clinical specimen. The overall study workflow is summarized in Fig 1.

**Fig 1:**
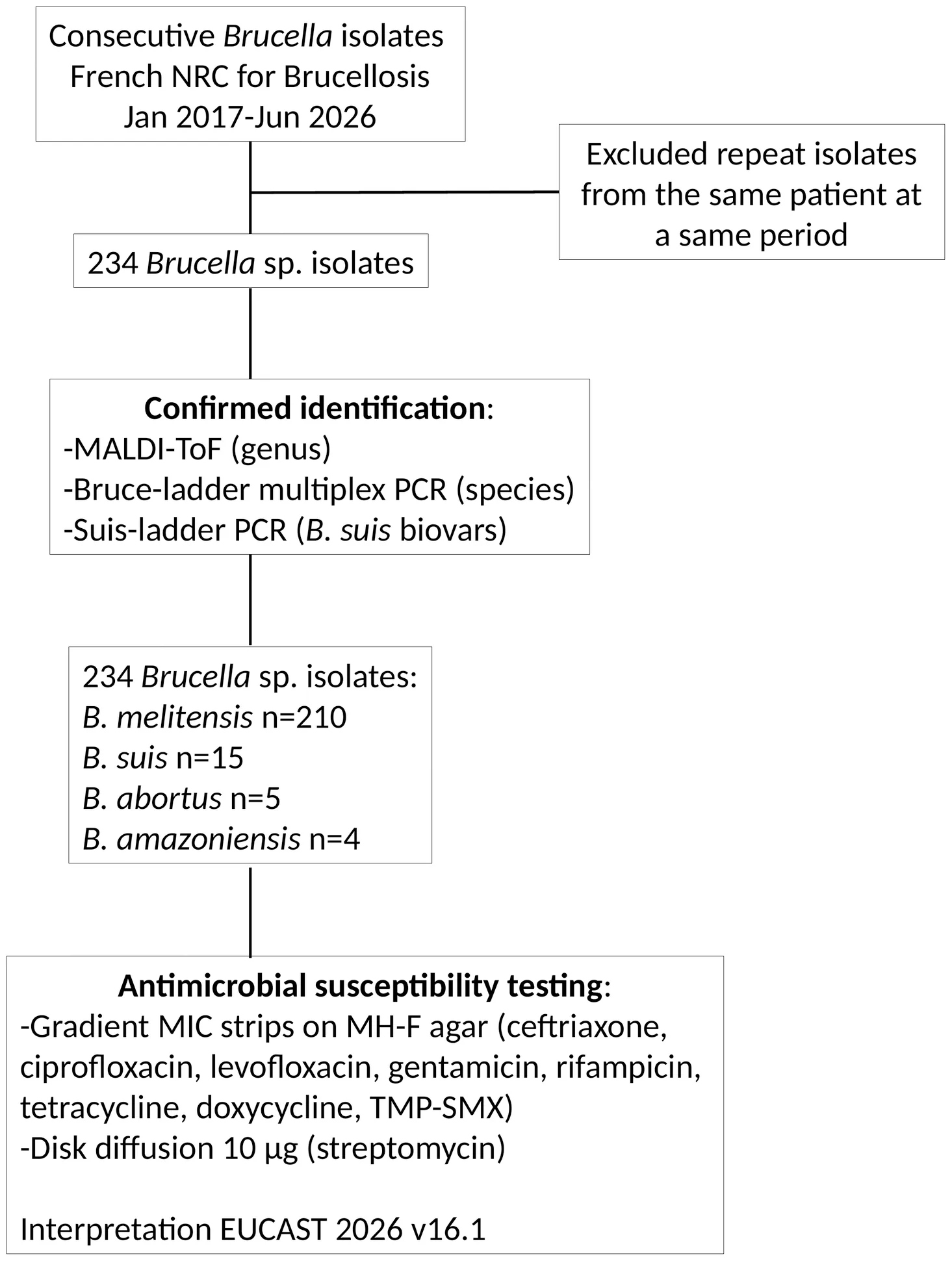
Study workflow. Flow diagram summarizing isolate reception at the French National Reference Centre for Brucellosis, inclusion and exclusion criteria, hierarchical species identification, antimicrobial susceptibility testing by gradient MIC strip and disk diffusion. MIC, minimum inhibitory concentration.

### Bacterial isolation and identification

Isolates were obtained from either clinical specimens (including blood, bone marrow, articular fluid aspirate, and cardiovascular tissue samples) or as pure cultures submitted by external laboratories. When multiple isolates from the same patient episode were received, only the first isolate was included in the analysis.

Bacterial identification was performed in a hierarchical manner (Fig 1). Genus-level identification was performed using MALDI-TOF mass spectrometry with the Bruker MALDI Biotyper system (Bruker Daltonics, Billerica, MA, USA) [16]. Species-level identification was confirmed by conventional multiplex PCR (Bruce-ladder) [17]. Further differentiation of *B. suis* biovars was achieved using an additional PCR assay (Suis-ladder) [18].

### Antimicrobial susceptibility testing

AST was performed using E-test strips (bioMérieux, Marcy-L’Etoile, France) for nine antibiotics: ceftriaxone, ciprofloxacin, levofloxacin, gentamicin, streptomycin, rifampicin, tetracycline, doxycycline, and trimethoprim-sulfamethoxazole. Inocula were prepared by adjusting bacterial suspensions to 0.5 McFarland turbidity using a SIRscan device (i2a, Montpellier, France) and inoculated onto cation-adjusted Mueller-Hinton agar supplemented with 5% defibrinated horse blood and 20 mg/L β-NAD (MH-F; Bio-Rad, Marnes-La-Coquette, France). E-test strips were applied to inoculated plates, and incubation was performed at 35 ± 1°C in 5% CO_2_ for 48 hours. For streptomycin, E-test results from the initial bioMérieux strips (Streptomycin SM164, Batch: 1010303960) were validated using parallel E-test strips from an alternative supplier (Streptomycin 92111, Batch: 71025023; i2a) and disk diffusion testing (10 μg disks; Bio-Rad). Inhibition zone diameters were measured using SIRweb software (i2a). All AST results were independently interpreted by two observers in accordance with EUCAST 2026 quality standards. MICs and inhibition zone diameters were interpreted using EUCAST 2026 clinical breakpoints for *Brucella melitensis* [14]. These breakpoints were defined from epidemiological cut-off (ECOFF) values, which represent the highest MIC (or smallest inhibition zone diameter) observed in *Brucella* isolates without phenotypically detectable acquired resistance mechanisms. For non-*melitensis* species lacking species-specific breakpoints, *B. melitensis* ECOFF values were applied as a conservative approach.

EUCAST clinical breakpoints and ECOFF values are formally derived from ISO 20776-1 broth microdilution. Broth microdilution is, however, poorly suited to routine work with *Brucella*, which is a slow-growing, fastidious Hazard Group 3 organism for which the preparation, inoculation and reading of microdilution panels both increase technical variability and substantially increase laboratory biorisk. Gradient MIC strips applied to MH-F agar were therefore deliberately selected, consistent with the stated aim of this study, which was to validate the EUCAST 2026 breakpoints under the conditions actually used by clinical laboratories rather than under reference conditions available only to specialized centres. Gradient diffusion has previously been shown to give high essential agreement with broth microdilution for *B. melitensis* [19]. Quality control was performed using reference strains *Streptococcus pneumoniae* ATCC 49619 and *Staphylococcus aureus* ATCC 29213.

### Statistical analysis

AST results were compared across the four *Brucella* species using non-parametric statistical methods. The Shapiro-Wilk test (α = 0.05) showed that MIC distributions departed from normality for most antibiotics; non-parametric tests were therefore used throughout. Global comparison of MICs among the four species was performed using the Kruskal-Wallis H test (α = 0.05), with pairwise comparisons conducted using the Mann-Whitney U test with Bonferroni correction (α = 0.0083 for six comparisons). Statistical significance was set at p < 0.05 for the global test and p < 0.0083 for pairwise comparisons after Bonferroni correction.

## Results

### Isolate collection and species distribution

Between January 2017 and June 2026, the French National Reference Centre for Brucellosis analyzed 234 consecutive *Brucella* isolates. Species identification revealed a marked predominance of *B. melitensis* (n = 210, 89.7%), followed by *Brucella suis* (n = 15, 6.4%)*, Brucella abortus* (n = 5, 2.1%)*,* and *Brucella amazoniensis* (n = 4, 1.7%). Within the *B. suis* collection, five isolates were identified as biovar 1 and ten as biovar 2. The majority of isolates were recovered from blood cultures (n = 116, 49.6%) and osteoarticular specimens (n = 22, 9.4%), while 88 strains (37.6%) were submitted as pure cultures by French laboratories and eight isolates (3.4%) were derived from other clinical specimens.

Geographically, the majority of strains originated from Africa (n = 153, 65.4%), with Algeria (n = 83) and Tunisia (n = 29) accounting for the highest proportion. A smaller subset was obtained from Europe (n = 29, 12.4%), including Portugal (n = 6), Italy (n = 4), and Spain (n = 1). France accounted for 23 isolates, comprising all *B. abortus* (n = 5), nine *B. suis* biovar 2 strains from mainland France, five *B. suis* biovar 1 from French Polynesia, and four *B. amazoniensis* from French Guiana. Twenty-nine isolates (12.4%) were acquired in the Middle East, predominantly from Turkey (n = 12) and Lebanon (n = 8). Eight isolates (3.4%) originated from Asia (including China, India, Afghanistan, Tibet, Nepal), and five (2.3%) from South America (four *B. amazoniensis* from French Guiana and one *B. suis* biovar 1 from Brazil). Detailed demographic and specimen source data are presented in S1 Table.

### Overall antimicrobial susceptibility testing results

The MICs distributions for eight antibiotics are presented in Tables 1 and S2. AST demonstrated excellent agreement with expected results. All quality control strains produced results within EUCAST 2026 acceptable ranges, confirming proper inoculum preparation, medium composition, incubation conditions, and technical execution of the methodology (S3 Table). MIC distribution for the eight tested antibiotics were as follows: ceftriaxone, 0.032 − 2 mg/L; ciprofloxacin, 0.125 − 0.5 mg/L; levofloxacin, 0.125 − 1 mg/L; gentamicin, 0.064 − 0.5 mg/L; tetracycline, 0.032 − 0.25 mg/L; doxycycline, 0.032 − 0.25 mg/L; rifampicin, 0.25 − 2 mg/L, and trimethoprim-sulfamethoxazole, 0.016 − 0.125 mg/L. According to EUCAST ECOFF values, all 234 isolates were classified as susceptible; no isolates were resistant to any tested antibiotics.

**Table 1:** MIC distribution for current antibiotics against *Brucella* isolates from French National Reference Centre of Brucellosis during 2017-2026 period.

Antibiotics	MICs (mg/L)								
	0.016	0.032	0.064	0.125	0.25	0.5	1	2	≥ 4
Ceftriaxone	0	1	2	9	60	144	16	2	0
Ciprofloxacin	0	0	0	3	52	179	0	0	0
Levofloxacin	0	0	0	3	54	156	21	0	0
Gentamicin	0	0	1	12	137	84	0	0	0
Rifampicin	0	0	0	0	7	26	85	116	0
Tetracycline	0	21	58	135	20	0	0	0	0
Doxycycline	0	17	57	140	20	0	0	0	0
TMP-SMX	20	127	51	36	0	0	0	0	0

TMP-SMX, trimethoprim-sulfamethoxazole; In grey, the ECOFF breakpoints defined by EUCAST for each antibiotic.

Table 1 illustrates the MIC distribution across all eight antibiotics. For most agents, MICs were well-separated from the ECOFF, indicating a clear categorization of the wild-type *Brucella* population. However, rifampicin demonstrated a distinctive pattern, with a substantial proportion of isolates exhibiting MICs in proximity to the ECOFF.

To highlight this clinically relevant finding, we analyzed the proportion of isolates exhibiting MICs within two dilutions of the ECOFF, a threshold generally considered microbiologically significant [20]. The following percentages were observed: ciprofloxacin (1 − 2 mg/L) and tetracycline (0.5 − 1 mg/L), 0%; ceftriaxone (2 − 4 mg/L), 0.9%; doxycycline (0.25 − 0.5 mg/L), 8.5%; levofloxacin (1 − 2 mg/L), 9.0%; trimethoprim-sulfamethoxazole (0.125 − 0.25 mg/L), 15.4%; gentamicin (0.5 − 1 mg/L), 35.9%, and rifampicin (2 − 4 mg/L), 49.6% (Table 1). This finding highlights that rifampicin demonstrated the highest proportion of isolates with MICs approaching the ECOFF, warranting continued surveillance for potential resistance development and suggesting the appropriateness of the EUCAST 2026 surveillance strategy for this agent.

### Species-specific antimicrobial susceptibility profiles

Susceptibility patterns were generally consistent across all *Brucella* species (S2 Table). Statistical analysis compared MIC distributions across the four *Brucella* species using non-parametric testing (Kruskal-Wallis H test with Bonferroni-corrected Mann-Whitney U pairwise comparisons). Seven of eight antibiotics (ceftriaxone, ciprofloxacin, gentamicin, levofloxacin, rifampicin, doxycycline and tetracycline) showed statistically significant differences in MIC distributions among species. Notably, trimethoprim-sulfamethoxazole was the only antibiotic demonstrating statistically homogeneous distributions across all four species (Kruskal-Wallis, p = 0.435).

*B. melitensis* (n = 210) demonstrated characteristically tight, predictable MIC distributions with modal values consistently at or near the lower susceptible range. This species exhibited the highest median MIC values for most antibiotics (ceftriaxone median 0.38 mg/L, ciprofloxacin 0.38 mg/L, levofloxacin 0.5 mg/L, rifampicin 1.5 mg/L, and tetracycline 0.094 mg/L), confirming its appropriateness as the reference species for EUCAST breakpoint development.

*B. suis* (n = 15) and *B. abortus* (n = 5) demonstrated broadly similar MIC profiles, with pairwise Mann-Whitney comparisons revealing no statistically significant differences for most antibiotics (p > 0.0083 with Bonferroni correction). Both species typically exhibited lower median MICs compared to *B. melitensis*. Notably, *B. suis* demonstrated the greatest variability in MIC values among the four species, particularly for rifampicin and levofloxacin, which may reflect strain-level heterogeneity or biovar-specific differences. *B. amazoniensis* (n = 4) demonstrated unique characteristics, particularly elevated cephalosporin MICs (median 0.875 mg/L for ceftriaxone) compared to *B. melitensis* (median 0.38 mg/L) and other species. However, pairwise Mann-Whitney comparisons between *B. amazoniensis* and all other species were not statistically significant after Bonferroni correction (α = 0.0083), likely due to the small sample size. Despite higher individual MICs, all values remained well within susceptible range per EUCAST 2026 breakpoints.

### Streptomycin susceptibility testing

E-test determination of streptomycin MICs (Streptomycin SM 1024; bioMérieux) yielded results that were discordant both with the disk diffusion data obtained on the same isolates and with the wild-type distribution expected for this genus. Among the first 150 isolates tested, 12 (8.0%) had streptomycin MICs above the EUCAST streptomycin ECOFF and would therefore have been reported as non-wild-type, while the distribution as a whole was displaced upwards relative to that obtained by disk diffusion (S1 Fig). To exclude batch-related artifacts, E-test strips from a second manufacturer (i2a), of a different production lot, were evaluated on the same 150 isolates. The elevated MICs were reproduced, excluding a lot- or manufacturer-specific defect. This pattern was consistent across all four *Brucella* species, arguing against a species-specific phenomenon and pointing instead to a potential *Brucella-*specific limitation of E-test methodology for streptomycin.

To validate streptomycin susceptibility, disk diffusion AST using 10 μg disks was performed on all 234 isolates. All evaluable isolates demonstrated clear, measurable inhibition zones ranging from 21 mm to 35 mm (Fig 2).

**Fig 2:**
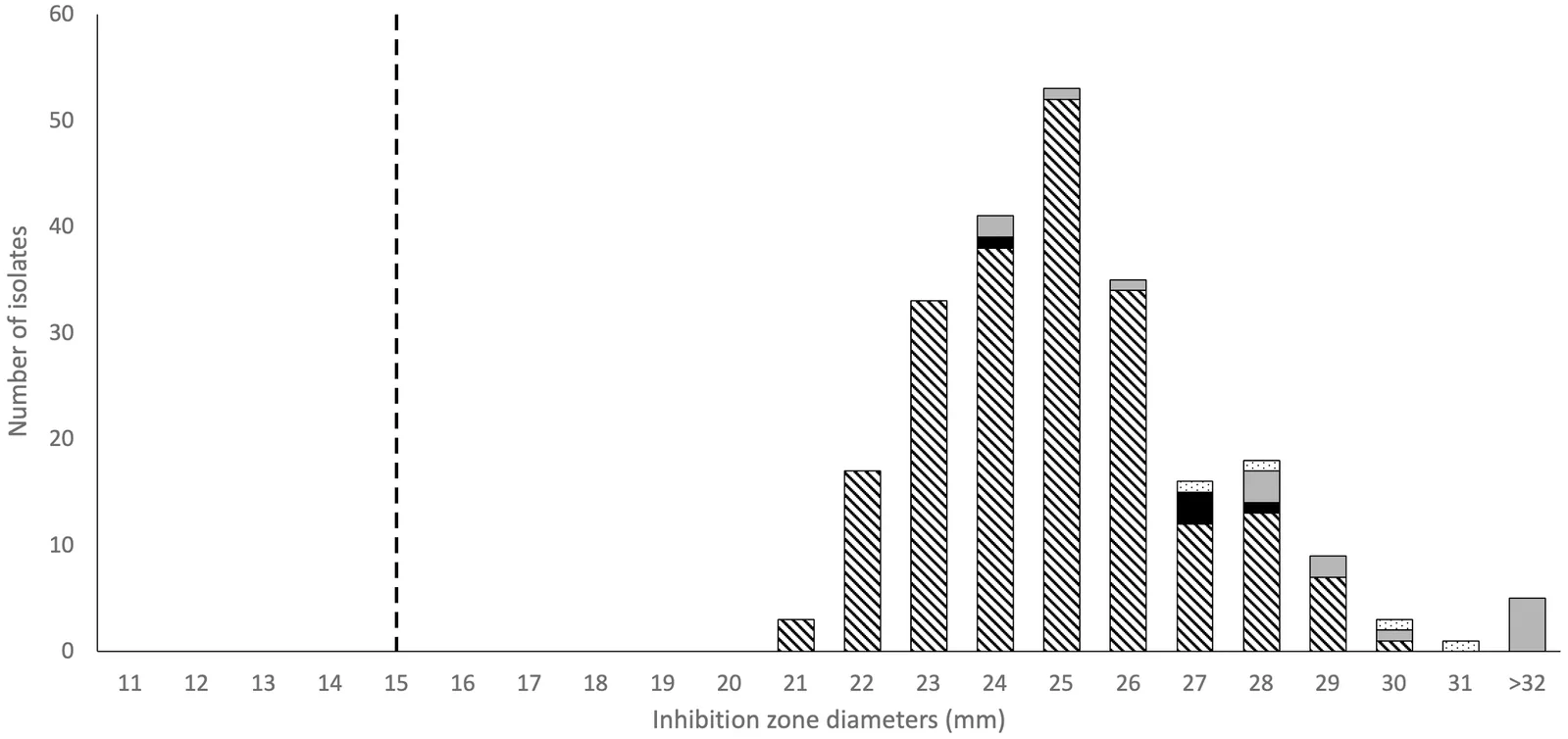
Streptomycin disk diffusion susceptibility of *Brucella* sp. isolates. Streptomycin susceptibility of 234 *Brucella* isolates determined by disk diffusion method (10 μg disks, zone diameter in mm). Species are represented as follows: hatched bars for *B. melitensis*, black bars for *B. abortus*, grey bars for *B. suis*, and dotted bars for *B. amazoniensis*. The EUCAST 2026 clinical breakpoint for susceptibility is indicated by a dashed line (≥15 mm = susceptible, < 15 mm = resistant).

Streptomycin disk diffusion zone diameters demonstrated statistically significant heterogeneity among the four *Brucella* species (Kruskal-Wallis H = 9.698, p = 0.021). *B. melitensis* showed the smallest median zone diameter (25 ± 1.8 mm, range 21–30 mm). *B. abortus* (median 27 ± 1.4, range 24–28 mm) and *B. suis* (median 28 ± 3.2 mm, range 24–35 mm) demonstrated larger zones. *B. amazoniensis* exhibited larger zones (median 29 ± 1.6 mm, range 27–31 mm). Mann-Whitney pairwise comparisons with Bonferroni correction revealed that *B. melitensis* differed significantly from *B. suis* (p < 0.001) and *B. amazoniensis* (p = 0.0018), while no significant differences were observed among the other species. Importantly, all 234 isolates remained well above the EUCAST 2026 susceptible breakpoint (≥15 mm), ensuring clinically reliable and consistent interpretation across all species.

## Discussion

Although *Brucella* spp. are generally considered susceptible to a broad range of antibiotics, increasing reports of antimicrobial resistance worldwide raise concerns about the long-term efficacy of standard treatment regimens [10–13]. A recent meta-analysis covering 59 studies with approximately 7,000 isolates reported global resistance rates of 3.4% (95% CI: 1.7–6.8%) for trimethoprim-sulfamethoxazole and 4.6% (95% CI: 2.7–7.7%) for rifampicin, with increasing temporal trends, particularly in *B. melitensis*. Geographically, resistance is highest in Africa, driven by antibiotic overuse and inconsistent treatment guidelines [11]. Resistance patterns vary substantially by region, species, and specimen origin, underscoring the critical importance of regular AST, even in non-endemic countries such as France, where population mobility (travel migration, occupational exposure) increases the risk of importing resistant strains. This emphasizes the need for standardized AST protocols and clearly defined clinical breakpoints to guide therapeutic decision-making.

The mechanisms of resistance in *Brucella* are complex and incompletely understood. Efflux pump systems, particularly RND-type (Resistance-Nodulation-Division) transporters, play major roles in resistance to tetracyclines, rifampicin, aminoglycosides, β-lactams, and fluoroquinolones [10–13]. Target site mutations contribute to resistance through alterations in the *rpoB* gene for rifampicin [11,21], *folA, folP* and *dhfr* genes for cotrimoxazole [11,22], and *gyrA* and *gyrB* for fluoroquinolones [12]. Recently, rifampicin resistance has been associated with mutations in the serine/threonine protein kinase (STPK)-encoding gene [23]. Enzymatic inactivation also contributes to resistance phenotypes, particularly through aminoglycoside-modifying enzymes that target streptomycin and gentamicin [13]. Additionally, the intracellular lifestyle of *Brucella* within macrophages and the role of VirB secretion system (particularly VirB7-11) limit antibiotic penetration, complicating treatment, increasing the risk of therapeutic failure, and promoting persistence and clinical relapse [24,25].

In our comprehensive analysis of 234 *Brucella* isolates collected between January 2017 and June 2026 from patients with diverse geographic origins (predominantly from Africa), all isolates demonstrated susceptibility to the main antibiotics used for brucellosis treatment, with no isolates classified as resistant according to EUCAST 2026 ECOFF values. This finding is reassuring and supports the continued clinical use of the recommended antimicrobial regimens for brucellosis treatment in France. Furthermore, our results validate the EUCAST 2026 ECOFF-based breakpoints for these key antibiotics.

Globally, resistance to fluoroquinolones remains relatively low, with meta-analytic resistance rates ranging from 1.6 to 4.6%, depending on the specific agent, although fluoroquinolone resistance is emerging in endemic regions [12]. Resistance to tetracyclines remains low worldwide (1.7%) [10], while resistance to third-generation cephalosporins is virtually absent, with only isolated cases reported in Iraq [9]. Aminoglycoside resistance is uncommon globally (2.3%), although higher rates (7%) have been reported in Turkey [13]. Streptomycin resistance has been documented in up to 2.7% of isolates, with Syria reporting the highest rate (49%) [13]. In comparison, our French collection showed no resistance to all tested agents, which is notably favorable compared to endemic regions but consistent with surveillance data from non-endemic European countries.

Rifampicin occupies a central role in *Brucella* therapy, and our analysis revealed an important epidemiological signal: 49.6% of isolates (n = 116) exhibited MICs within two dilutions of the EUCAST rifampicin ECOFF (MICs of 2–4 mg/L). Although all isolates remained susceptible per EUCAST breakpoints (≤2 mg/L for wild-type), this high proportion of MICs proximal to the breakpoint warrants ongoing surveillance. Notably, the Clinical and Laboratory Standards Institute (CLSI) employs a different rifampicin classification: susceptible MICs ≤ 1 mg/L, intermediate 2–3 mg/L, and resistant ≥4 mg/L [20]. Under CLSI criteria, a subset of our isolates would be classified as intermediate. Our data suggest that the EUCAST breakpoint of ≤2 mg/L remains clinically appropriate, as rifampicin is systematically included in brucellosis treatment regimens and clinical outcomes have been favorable in our cohort (notably no relapse under rifampicin treatment). However, continued monitoring for potential emergence of reduced susceptibility phenotypes is prudent.

A critical methodological discovery emerged from streptomycin testing. E-test strips initially suggested elevated MICs rate (8%). However, disk diffusion testing performed on all 234 isolates revealed clear, measurable inhibition zones ranging from 21 mm to 35 mm, with all isolates (100%) demonstrating susceptibility per EUCAST 2026 disk diffusion criteria (≥15 mm) [14]. These findings suggest a *Brucella*-specific limitation of E-test methodology for streptomycin, likely representing false MIC elevations due to technical artifacts rather than true resistance.

This interpretation is supported by the earliest comparative evaluation of gradient diffusion in *B. melitensis*, in which streptomycin, together with ofloxacin, showed the lowest essential agreement with broth microdilution of all agents examined (82%), whereas doxycycline reached complete agreement (100%) [19]. A plausible physicochemical explanation is that streptomycin is a highly polar, poorly diffusing aminoglycoside whose migration from the cellulose strip into MH-F agar is further impaired by the horse blood and β-NAD supplements required to support *Brucella* growth, generating a truncated diffusion gradient and a falsely elevated apparent MIC. Disk diffusion, in which the antimicrobial is released from a porous reservoir over a shorter radial distance, appears far less susceptible to this effect. Consequently, disk diffusion is strongly recommended as the reference method for streptomycin susceptibility testing in *Brucella* species. Notably, streptomycin susceptibility was preserved across the entire French national collection, confirming that streptomycin remains an effective agent for brucellosis treatment in France and supporting its continued role in complex or refractory cases. Moreover, streptomycin disk diffusion zone diameters demonstrated statistically significant heterogeneity among the four *Brucella* species (Kruskal-Wallis H = 9.698, p = 0.021). This finding indicates species-specific variations in streptomycin susceptibility that warrant attention for surveillance purposes.

Despite species-specific MIC variations and significant heterogeneity in MIC distributions among the four *Brucella* species for seven antibiotics (Kruskal-Wallis H test, p < 0.001–0.0092), all isolates remained universally susceptible to all tested antibiotics by EUCAST 2026 breakpoints. The use of *B. melitensis* EUCAST breakpoints for non-*melitensis* species remains justified, as these other species typically demonstrate lower MICs (greater safety margin). Interestingly, the emerging *B. amazoniensis*, despite elevated MICs for some agents, still maintains adequate susceptibility.

These findings, generated in a country where brucellosis is almost entirely imported, carry implications well beyond France. Because 65.4% of our isolates were acquired in Africa and 12.4% in the Middle East, the collection is in practice a window onto the populations circulating in endemic regions where systematic AST is rarely feasible. The streptomycin finding matters most in those same settings, where gradient strips are preferred over broth microdilution for their simplicity: an uncorrected artefact would generate spurious reports of aminoglycoside resistance and distort the regional estimates that are themselves built from aggregated laboratory reports. That method choice alone can move an apparent resistance rate from 0% to 8% argues for treating AST harmonization as a surveillance priority in its own right.

Several priorities follow directly from these results. The streptomycin discrepancy should be quantified against ISO 20776-1 broth microdilution in a multicentre study, so that EUCAST can issue either a method-specific correction factor or a formal recommendation for this agent. Whole-genome sequencing of the isolates with rifampicin MICs adjacent to the ECOFF would establish whether *rpoB* or serine/threonine protein kinase polymorphisms already circulate at low frequency. Finally, species-specific ECOFFs for the non-*melitensis* species will require pooled international collections, since no single national centre accumulates sufficient numbers.

This study has several limitations. First, *B. abortus* and *B. amazoniensis* are represented by small sample sizes (n = 5 and 4, respectively), limiting statistical power for species-specific comparisons and conclusions. Second, the collection is geographically biased, with 65.4% of isolates originating from Africa, primarily from Algeria; consequently, our findings may not fully represent global *Brucella* susceptibility patterns. Third, MICs were determined by gradient diffusion on MH-F agar rather than by ISO 20776–1 broth microdilution, the reference method from which EUCAST breakpoints are formally derived. Although this was a deliberate choice consistent with the aim of validating the breakpoints under routine conditions, method-dependent shifts in MIC of one doubling dilution cannot be excluded for individual agents, and the streptomycin data show that such shifts may occasionally be larger. Finally, the data reflect only the French National Reference Centre’s experience and may not be generalizable to all European or non-endemic settings.

## Conclusion

This comprehensive analysis of 234 *Brucella* isolates supports three practical conclusions. The EUCAST 2026 breakpoints are workable in a routine clinical laboratory and correctly classify a fully wild-type national collection, which is the first condition any new breakpoint must satisfy. The method used to generate the MIC, however, is not neutral: for streptomycin, gradient diffusion and disk diffusion assign different susceptibility categories to the same organism, and only disk diffusion agrees with the clinical and epidemiological evidence; disk diffusion should be specified as the method of choice for this agent. The concentration of rifampicin MICs immediately below the ECOFF defines a measurable surveillance target rather than a present clinical problem, and it is the parameter most likely to change first should resistance begin to emerge in this genus. Together these findings justify continued use of *B. melitensis*-based EUCAST 2026 breakpoints for all *Brucella* species and highlight the importance of geographic-specific surveillance and method standardization for optimal brucellosis management.

## Supporting information

S1 Fig Streptomycin E-test MIC determination. Streptomycin minimum inhibitory concentration (MIC) values (mg/L) for 234 *Brucella* isolates determined using E-test strips (Streptomycin SM 1024; bioMérieux). Species are represented as follows: hatched bars for *B. melitensis*, black bars for *B. abortus*, grey bars for *B. suis*, and dotted bars for *B. amazoniensis*. The EUCAST 2026 clinical breakpoint for susceptibility is indicated by a dashed line (≤1 mg/L = susceptible, > 1 mg/L = resistant).
(TIFF)

S1 Table Isolate origins and bacteriological characteristics. Detailed demographic data for all 234 *Brucella* isolates analyzed, including: geographic origin, specimen source, and *Brucella* species identification. Data are presented sequentially by isolate number.
(DOCX)

S2 Table MIC distributions by *Brucella* species. Antimicrobial susceptibility data for eight antibiotics tested by E-test, stratified by *Brucella* species.
(DOCX)

S3 Table Quality control results for EUCAST 2026 breakpoints. For each antibiotic, quality control values established on *Staphylococcus aureus* ATCC 29213 and *Streptococcus pneumoniae* ATCC 49619 are presented.
(DOCX)
