# Supplementary material for: Validation of EUCAST 2026 breakpoints for Brucella species using routine laboratory methods: A comprehensive French National Reference Centre study
Source: PLoS Negl Trop Dis. 2026 Oct 27;20(10):e0014817. doi: 10.1371/journal.pntd.0014817 (PMC13712602; doi:10.1371/journal.pntd.0014817)
Supplement: Supplementary file 1 [file pntd.0014817.s002.docx]

**S1 Table.** **Origin and bacteriological characteristics of the 234 *Brucella* isolates analysed**.

| **Isolate  ID** | **Date of receipt** | **Species and biovars** | **Type**  **of sampling** | **Stay in high-risk countries** |
| --- | --- | --- | --- | --- |
| BRSO-2017-001 | April 2017 | *B. melitensis* | Strain | India |
| BRSO-2017-004 | August 2017 | *B. melitensis* | Blood culture | Tunisia |
| BRSO-2017-005 | August 2017 | *B. melitensis* | Blood culture | Maghreb |
| BRSO-2017-009 | August 2017 | *B. melitensis* | Blood culture | Algeria |
| BRSO-2017-011 | August 2017 | *B. melitensis* | Strain | Kuwait |
| BRSO-2017-013 | September 2017 | *B. melitensis* | Strain | Tunisia |
| BRSO-2017-015 | September 2017 | *B. melitensis* | Strain | Ethiopia |
| BRSO-2017-016 | August 2017 | *B. melitensis* | Strain | Algeria |
| BRSO-2017-019 | June 2017 | *B. melitensis* | Strain | Maghreb |
| BRSO-2017-020 | July 2017 | *B. melitensis* | Strain | China |
| BRSO-2017-022 | November 2017 | *B. suis bv1* | Blood culture | Brazil |
| BRSO-2017-024 | November 2017 | *B. melitensis* | Blood culture | Tunisia |
| BRSO-2017-026 | December 2017 | *B. melitensis* | Blood culture | Maghreb |
| BRSO-2017-028 | December 2017 | *B. melitensis* | Blood culture | Algeria |
| BRSO-2017-030 | December 2017 | *B. melitensis* | Blood culture | Tunisia |
| BRSO-2018-036 | February 2018 | *B. melitensis* | Synovial Fluid | Tunisia |
| BRSO-2018-043 | April 2018 | *B. melitensis* | Strain | Egypt |
| BRSO-2018-045 | April 2018 | *B. abortus* | Strain | France |
| BRSO-2018-048 | May 2018 | *B. melitensis* | Blood culture | Algeria |
| BRSO-2018-049 | May 2018 | *B. melitensis* | Blood culture | Algeria |
| BRSO-2018-050 | May 2018 | *B. melitensis* | Blood culture | Algeria |
| BRSO-2018-051 | June 2018 | *B. melitensis* | Blood culture | Kuwait |
| BRSO-2018-055 | July 2018 | *B. melitensis* | Blood culture | Tunisia |
| BRSO-2018-056 | August 2018 | *B. melitensis* | Blood culture | Tunisia |
| BRSO-2018-057 | July 2018 | *B. melitensis* | Blood culture | Algeria |
| BRSO-2018-063 | September 2018 | *B. melitensis* | Blood culture | Tibet |
| BRSO-2018-064 | October 2018 | *B. melitensis* | Blood culture | Saudi Arabia |
| BRSO-2018-065 | October 2018 | *B. melitensis* | Blood culture | Algeria |
| BRSO-2018-066 | October 2018 | *B. melitensis* | Blood culture | France (Laboratory contamination) |
| BRSO-2018-068 | October 2018 | *B. melitensis* | Blood culture | Algeria |
| BRSO-2018-070 | November 2018 | *B. melitensis* | Synovial Fluid | Maghreb |
| BRSO-2018-073 | November 2018 | *B. melitensis* | Blood culture | Tunisia (Relapse) |
| BRSO-2018-074 | November 2018 | *B. melitensis* | Blood culture | Lebanon |
| BRSO-2018-075 | November 2018 | *B. melitensis* | Strain | Maghreb |
| BRSO-2018-077 | December 2018 | *B. melitensis* | Blood culture | Algeria |
| BRSO-2018-078 | December 2018 | *B. melitensis* | Strain | Maghreb |
| BRSO-2018-080 | December 2018 | *B. melitensis* | Blood culture | Algeria |
| BRSO-2019-081 | January 2019 | *B. melitensis* | Vertebral biopsy | Algeria |
| BRSO-2019-087 | February 2019 | *B. melitensis* | Blood culture | Morocco |
| BRSO-2019-092 | March 2019 | *B. melitensis* | Blood culture | France (Laboratory contamination) |
| BRSO-2019-093 | March 2019 | *B. melitensis* | Blood culture | Algeria |
| BRSO-2019-094 | March 2019 | *B. melitensis* | Vertebral biopsy | Algeria |
| BRSO-2019-095 | March 2019 | *B. melitensis* | Synovial Fluid | Maghreb |
| BRSO-2019-097 | April 2019 | *B. melitensis* | Blood culture | Portugal |
| BRSO-2019-100 | April 2019 | *B. melitensis* | Vertebral biopsy | Algeria |
| BRSO-2019-102 | May 2019 | *B. melitensis* | Blood culture | Portugal |
| BRSO-2019-103 | May 2019 | *B. melitensis* | Synovial Fluid | Portugal |
| BRSO-2019-104 | May 2019 | *B. melitensis* | Vertebral biopsy | Algeria |
| BRSO-2019-105 | May 2019 | *B. melitensis* | Blood culture | Portugal |
| BRSO-2019-106 | May 2019 | *B. melitensis* | Blood culture | Maghreb |
| BRSO-2019-112 | June 2019 | *B. melitensis* | Blood culture | France (Laboratory contamination) |
| BRSO-2019-114 | June 2019 | *B. melitensis* | Blood culture | Algeria |
| BRSO-2019-117 | June 2019 | *B. melitensis* | Blood culture | Morocco |
| BRSO-2019-120 | July 2019 | *B. melitensis* | Blood culture | Tunisia |
| BRSO-2019-121 | July 2019 | *B. melitensis* | Synovial Fluid | Maghreb |
| BRSO-2019-122 | July 2019 | *B. melitensis* | Blood culture | Turkey |
| BRSO-2019-124 | July 2019 | *B. melitensis* | Blood culture | Turkey |
| BRSO-2019-125 | July 2019 | *B. melitensis* | Blood culture | Morocco |
| BRSO-2019-127 | July 2019 | *B. melitensis* | Blood culture | Algeria |
| BRSO-2019-129 | July 2019 | *B. melitensis* | Blood culture | Morocco |
| BRSO-2019-130 | August 2019 | *B. melitensis* | Synovial Fluid | Maghreb |
| BRSO-2019-135 | August 2019 | *B. melitensis* | Blood culture | Maghreb |
| BRSO-2019-139 | August 2019 | *B. melitensis* | Blood culture | Algeria |
| BRSO-2019-143 | September 2019 | *B. melitensis* | Knee biopsy | Portugal-Spain |
| BRSO-2019-149 | September 2019 | *B. melitensis* | Gastric fluid | Algeria |
| BRSO-2019-152 | October 2019 | *B. melitensis* | Blood culture | Italia (Sicily) |
| BRSO-2019-155 | October 2019 | *B. melitensis* | Blood culture | Afghanistan |
| BRSO-2019-156 | October 2019 | *B. melitensis* | Blood culture | Lebanon |
| BRSO-2019-157 | November 2019 | *B. suis bv2* | Knee biopsy | France |
| BRSO-2019-158 | November 2019 | *B. melitensis* | Blood culture | Algeria |
| BRSO-2019-159 | November 2019 | *B. melitensis* | Blood culture | Algeria |
| BRSO-2020-169 | January 2020 | *B. melitensis* | Strain | Maghreb |
| BRSO-2020-170 | February 2020 | *B. melitensis* | Strain | Algeria |
| BRSO-2020-179 | March 2020 | *B. melitensis* | Defibrillator lead | Maghreb |
| BRSO-2020-181 | March 2020 | *B. melitensis* | Strain | Maghreb |
| BRSO-2020-182 | March 2020 | *B. melitensis* | Blood culture | Maghreb |
| BRSO-2020-191 | June 2020 | *B. melitensis* | Strain | Algeria |
| BRSO-2020-195 | June 2020 | *B. melitensis* | Strain | Algeria |
| BRSO-2020-196 | June 2020 | *B. melitensis* | Strain | Algeria |
| BRSO-2020-197 | June 2020 | *B. melitensis* | Blood culture | Maghreb |
| BRSO-2020-200 | June 2020 | *B. melitensis* | Strain | Maghreb |
| BRSO-2020-209 | August 2020 | *B. abortus* | Blood culture | Italy |
| BRSO-2020-210 | August 2020 | *B. melitensis* | Blood culture | Algeria |
| BRSO-2020-211 | August 2020 | *B. melitensis* | Blood culture | Tunisia |
| BRSO-2020-213 | September 2020 | *B. amazoniensis* | Blood culture | French Guiana |
| BRSO-2020-214 | December 2020 | *B. suis* *bv* *2* | Aortic aneurysm biopsy | France |
| BRSO-2020-215 | December 2020 | *B. abortus* | Strain | France (relapse) |
| BRSO-2021-216 | January 2021 | *B. melitensis* | Blood culture | Algeria |
| BRSO-2021-217 | January 2021 | *B. melitensis* | Blood culture | Maghreb |
| BRSO-2021-218 | January 2021 | *B. melitensis* | Blood culture | Algeria |
| BRSO-2021-219 | January 2021 | *B. suis bv 2* | Anterior hip capsule | France |
| BRSO-2021-220 | February 2021 | *B. melitensis* | Blood culture | Lebanon |
| BRSO-2021-221 | February 2021 | *B. suis bv 1* | Breast biopsy | French Polynesia |
| BRSO-2021-230 | March 2021 | *B. amazoniensis* | Vertebral biopsy | French Guiana |
| BRSO-2021-231 | March 2021 | *B. melitensis* | Liver biopsy | Algeria |
| BRSO-2021-235 | April 2021 | *B. melitensis* | Blood culture | Maghreb |
| BRSO-2021-243 | May 2021 | *B. melitensis* | Strain | Maghreb |
| BRSO-2021-244 | May 2021 | *B. melitensis* | Blood culture | Israel |
| BRSO-2021-250 | June 2021 | *B. melitensis* | Blood culture | Lebanon |
| BRSO-2021-255 | September 2021 | *B. melitensis* | Synovial Fluid | Italy |
| BRSO-2021-256 | September 2021 | *B. melitensis* | Strain | Algeria |
| BRSO-2021-257 | September 2021 | *B. suis bv 2* | Abscess | France |
| BRSO-2021-260 | September 2021 | *B. melitensis* | Strain | Chad |
| BRSO-2021-262 | September 2021 | *B. melitensis* | Blood culture | Israel |
| BRSO-2021-263 | September 2021 | *B. melitensis* | Strain | Tunisia |
| BRSO-2021-266 | October 2021 | *B. melitensis* | Whole blood | Kuwait |
| BRSO-2021-267 | October 2021 | *B. melitensis* | Blood culture | Tunisia |
| BRSO-2021-269 | November 2021 | *B. melitensis* | Blood culture | Algeria |
| BRSO-2021-272 | November 2021 | *B. melitensis* | Strain | Tunisia |
| BRSO-2021-273 | November 2021 | *B. melitensis* | Blood culture | Lebanon |
| BRSO-2021-280 | December 2021 | *B. melitensis* | Vertebral biopsy | Tunisia |
| BRSO-2021-281 | December 2021 | *B. melitensis* | Vertebral biopsy | Unknown |
| BRSO-2022-283 | July 2022 | *B. melitensis* | Blood culture | Algeria |
| BRSO-2022-284 | July 2022 | *B. melitensis* | Strain | Italy-Tunisia |
| BRSO-2022-302 | August 2022 | *B. melitensis* | Blood culture | Algeria |
| BRSO-2022-305 | April 2022 | *B. melitensis* | Strain | Tunisia |
| BRSO-2022-306 | April 2022 | *B. melitensis* | Blood culture | Djibouti |
| BRSO-2022-307 | March 2022 | *B. melitensis* | Strain | Armenia |
| BRSO-2022-314 | February 2022 | *B. melitensis* | Strain | Algeria |
| BRSO-2022-315 | February 2022 | *B. melitensis* | Blood culture | Algeria |
| BRSO-2022-316 | August 2022 | *B. melitensis* | Blood culture | Algeria |
| BRSO-2022-318 | October 2022 | *B. melitensis* | Blood culture | Turkey |
| BRSO-2022-321 | June 2022 | *B. melitensis* | Blood culture | Algeria |
| BRSO-2022-324 | June 2022 | *B. melitensis* | Blood culture | Algeria |
| BRSO-2022-326 | June 2022 | *B. melitensis* | Strain | Algeria |
| BRSO-2022-328 | June 2022 | *B. melitensis* | Vertebral biopsy | Turkey |
| BRSO-2022-329 | June 2022 | *B. melitensis* | Strain | Turkey |
| BRSO-2022-334 | July 2022 | *B. melitensis* | Strain | Tunisia-Dubai |
| BRSO-2022-335 | July 2022 | *B. melitensis* | Blood culture | Algeria |
| BRSO-2022-337 | July 2022 | *B. melitensis* | Strain | Algeria |
| BRSO-2022-340 | January 2022 | *B. melitensis* | Blood culture | Algeria |
| BRSO-2022-344 | August 2022 | *B. melitensis* | Blood culture | Maghreb |
| BRSO-2022-347 | August 2022 | *B. melitensis* | Blood culture | Algeria |
| BRSO-2022-348 | August 2022 | *B. melitensis* | Blood culture | Algeria |
| BRSO-2022-349 | August 2022 | *B. melitensis* | Strain | Algeria |
| BRSO-2022-350 | August 2022 | *B. suis bv 1* | Strain | Wallis and Futuna |
| BRSO-2022-351 | August 2022 | *B. melitensis* | Blood culture | Lebanon |
| BRSO-2022-355 | September 2022 | *B. melitensis* | Blood culture | Algeria |
| BRSO-2022-367 | September 2022 | *B. melitensis* | Blood culture | Algeria |
| BRSO-2022-368 | September 2022 | *B. melitensis* | Strain | Algeria |
| BRSO-2022-371 | September 2022 | *B. melitensis* | Blood culture | Maghreb |
| BRSO-2022-373 | October 2022 | *B. melitensis* | Blood culture | Algeria |
| BRSO-2022-375 | October 2022 | *B. melitensis* | Blood culture | Algeria |
| BRSO-2022-382 | October 2022 | *B. melitensis* | Blood culture | Turkey |
| BRSO-2022-383 | February 2022 | *B. melitensis* | Blood culture | Algeria |
| BRSO-2022-388 | November 2022 | *B. abortus* | Strain | France |
| BRSO-2022-389 | November 2022 | *B. melitensis* | Blood culture | Maghreb |
| BRSO-2022-395 | September 2022 | *B. melitensis* | Strain | China |
| BRSO-2022-398 | December 2022 | *B. melitensis* | Strain | Lebanon |
| BRSO-2022-399 | December 2022 | *B. melitensis* | Blood culture | Djibouti |
| BRSO-2023-424 | April 2023 | *B. melitensis* | Blood culture | Jordan |
| BRSO-2023-436 | June 2023 | *B. melitensis* | Strain | Algeria |
| BRSO-2023-456 | February 2023 | *B. melitensis* | Strain | Algeria |
| BRSO-2023-457 | August 2023 | *B. melitensis* | Blood culture | Algeria |
| BRSO-2023-458 | August 2023 | *B. melitensis* | Blood culture | Maghreb |
| BRSO-2023-460 | August 2023 | *B. melitensis* | Blood culture | Maghreb |
| BRSO-2023-470 | October 2023 | *B. melitensis* | Blood culture | Turkey-Algeria |
| BRSO-2023-471 | October 2023 | *B. melitensis* | Blood culture | Maghreb |
| BRSO-2023-475 | July 2023 | *B. melitensis* | Blood culture | Spain |
| BRSO-2023-482 | October 2023 | *B. melitensis* | Blood culture | Algeria |
| BRSO-2023-484 | November 2023 | *B. melitensis* | Blood culture | Maghreb |
| BRSO-2023-486 | November 2023 | *B. melitensis* | Blood culture | Lebanon |
| BRSO-2024-502 | February 2024 | *B. melitensis* | Strain | Turkey |
| BRSO-2024-507 | February 2024 | *B. melitensis* | Strain | Egypt-Tunisia |
| BRSO-2024-512 | March 2024 | *B. melitensis* | Blood culture | Nepal |
| BRSO-2024-525 | March 2024 | *B. melitensis* | Blood culture | Algeria |
| BRSO-2024-534 | May 2024 | *B. melitensis* | Strain | Algeria |
| BRSO-2024-538 | June 2024 | *B. suis bv 1* | Aortic aneurysm biopsy | French Polynesia |
| BRSO-2024-540 | June 2024 | *B. melitensis* | Blood culture | Maghreb |
| BRSO-2024-541 | June 2024 | *B. melitensis* | Blood culture | Tunisia |
| BRSO-2024-543 | June 2024 | *B. melitensis* | Blood culture | Morocco |
| BRSO-2024-545 | June 2024 | *B. melitensis* | Blood culture | Turkey |
| BRSO-2024-548 | June 2024 | *B. melitensis* | Blood culture | Saudi Arabia |
| BRSO-2024-557 | July 2024 | *B. melitensis* | Blood culture | Portugal-Algeria |
| BRSO-2024-559 | July 2024 | *B. melitensis* | Strain | Tunisia |
| BRSO-2024-568 | September 2024 | *B. melitensis* | Strain | France (Laboratory contamination) |
| BRSO-2024-572 | September 2024 | *B. melitensis* | Blood culture | Algeria |
| BRSO-2024-576 | October 2024 | *B. melitensis* | Blood culture | Maghreb |
| BRSO-2024-578 | October 2024 | *B. melitensis* | Strain | Algeria |
| BRSO-2024-589 | November 2024 | *B. melitensis* | Strain | Algeria |
| BRSO-2024-594 | December 2024 | *B. melitensis* | Strain | Algeria |
| BRSO-2025-600 | February 2025 | *B. suis bv2* | Strain | France |
| BRSO-2025-601 | February 2025 | *B. melitensis* | Strain | Maghreb |
| BRSO-2025-602 | February 2025 | *B. melitensis* | Strain | Algeria |
| BRSO-2025-604 | February 2025 | *B. melitensis* | Strain | France (relapse) |
| BRSO-2025-615 | April 2025 | *B. melitensis* | Strain | Algeria |
| BRSO-2025-617 | April 2025 | *B. melitensis* | Blood culture | Algeria (relapse) |
| BRSO-2025-618 | April 2025 | *B. melitensis* | Strain | Tunisia |
| BRSO-2025-620 | April 2025 | *B. melitensis* | Strain | Tunisia |
| BRSO-2025-621 | April 2025 | *B. abortus* | Strain | France |
| BRSO-2025-624 | April 2025 | *B. melitensis* | Strain | Maghreb |
| BRSO-2025-626 | May 2025 | *B. melitensis* | Strain | Tunisia |
| BRSO-2025-627 | May 2025 | *B. melitensis* | Strain | Tunisia |
| BRSO-2025-628 | May 2025 | *B. melitensis* | Strain | Tunisia |
| BRSO-2025-631 | May 2025 | *B. melitensis* | Blood culture | Algeria |
| BRSO-2025-637 | June 2025 | *B. amazoniensis* | Strain | French Guyana |
| BRSO-2025-638 | June 2025 | *B. melitensis* | Blood culture | Djibouti |
| BRSO-2025-639 | June 2025 | *B. melitensis* | Strain | Jordania |
| BRSO-2025-640 | June 2025 | *B. melitensis* | Blood culture | Algeria |
| BRSO-2025-641 | July 2025 | *B. melitensis* | Blood culture | Algeria |
| BRSO-2025-647 | July 2025 | *B. melitensis* | Strain | Turkey |
| BRSO-2025-649 | July 2025 | *B. melitensis* | Strain | Maghreb |
| BRSO-2025-652 | July 2025 | *B. melitensis* | Strain | Algeria |
| BRSO-2025-653 | July 2025 | *B. melitensis* | Strain | Algeria |
| BRSO-2025-655 | August 2025 | *B. melitensis* | Blood culture | Algeria |
| BRSO-2025-662 | September 2025 | *B. melitensis* | Bone biopsy | Maghreb |
| BRSO-2025-665 | September 2025 | *B. amazoniensis* | Strain | French Guyana |
| BRSO-2025-671 | September 2025 | *B. suis bv1* | Strain | French Polynesia |
| BRSO-2025-676 | November 2025 | *B. melitensis* | Strain | Algeria |
| BRSO-2025-679 | November 2025 | *B. melitensis* | Blood culture | Algeria |
| BRSO-2025-684 | November 2025 | *B. melitensis* | Vertebral biopsy | Algeria |
| BRSO-2025-685 | November 2025 | *B. melitensis* | Strain | Algeria |
| BRSO-2025-689 | November 2025 | *B. melitensis* | Strain | Turkey |
| BRSO-2025-698 | December 2025 | *B. melitensis* | Strain | Marocco |
| BRSO-2026-705 | January 2026 | *B. melitensis* | Strain | Algeria |
| BRSO-2026-709 | January 2026 | *B. melitensis* | Articular fluid aspirate | Algeria |
| BRSO-2026-724 | February 2026 | *B. suis bv1* | Strain | French Polynesia |
| BRSO-2026-729 | February 2026 | *B. melitensis* | Strain | Tunisia |
| BRSO-2026-731 | March 2026 | *B. melitensis* | Strain | Algeria |
| BRSO-2026-737 | March 2026 | *B. melitensis* | Blood culture | Algeria |
| BRSO-2026-738 | March 2026 | *B. melitensis* | Strain | India |
| BRSO-2026-743 | March 2026 | *B. melitensis* | Strain | Maghreb |
| BRSO-2026-748 | April 2026 | *B. melitensis* | Blood culture | Turkey |
| BRSO-2026-750 | April 2026 | *B. melitensis* | Strain | Tunisia |
| BRSO-2026-753 | April 2026 | *B. melitensis* | Strain | Algeria |
| BRSO-2026-754 | April 2026 | *B. melitensis* | Strain | Algeria |
| BRSO-2026-755 | April 2026 | *B. melitensis* | Strain | Algeria |
| BRSO-2026-759 | May 2026 | *B. suis bv2* | Strain | France |
| BRSO-2026-760 | May 2026 | *B. melitensis* | Vertebral biopsy | Algeria (relapse) |
| BRSO-2026-761 | June 2026 | *B. suis bv2* | Strain | France |
| BRSO-2026-764 | June 2026 | *B. suis bv2* | Strain | France |
| BRSO-2026-765 | June 2026 | *B. melitensis* | Strain | Marocco |
| BRSO-2026-766 | June 2026 | *B. melitensis* | Strain | Maghreb |
| BRSO-2026-775 | June 2026 | *B. suis bv2* | Strain | France |

bv, biova
