# Supplementary material for: Validation of EUCAST 2026 breakpoints for Brucella species using routine laboratory methods: A comprehensive French National Reference Centre study
Source: PLoS Negl Trop Dis. 2026 Oct 27;20(10):e0014817. doi: 10.1371/journal.pntd.0014817 (PMC13712602; doi:10.1371/journal.pntd.0014817)
Supplement: Supplementary file 2 [file pntd.0014817.s003.docx]

**S2 Table.** **Detailed MICs distribution for each antibiotic, stratified by *Brucella* species**.

| **Antibiotics** | **MICs (mg/L)** | | | | | | | | | **Kruskal-Wallis H, P ^a^** | |
| --- | --- | --- | --- | --- | --- | --- | --- | --- | --- | --- | --- |
| **/ Species** | **0.016** | **0.032** | **0.064** | **0.125** | **0.25** | **0.5** | **1** | **2** | **≥ 4** |  |  |
| **Ceftriaxone** |  | | | | | | | | |  |  |
| Total (234) | 0 | 1 | 2 | 9 | 60 | 144 | 16 | 2 | 0 | 29.427, <0.001 |  |
| *B. melitensis* (210) | 0 | 0 | 0 | 4 | 52 | 141 | 11 | 2 | 0 |  |  |
| *B. suis* (15) | 0 | 0 | 1 | 5 | 6 | 1 | 2 | 0 | 0 |  |  |
| *B. abortus* (5) | 0 | 1 | 1 | 0 | 2 | 1 | 0 | 0 | 0 |  |  |
| *B. amazoniensis* (4) | 0 | 0 | 0 | 0 | 0 | 1 | 3 | 0 | 0 |  |  |
| **Ciprofloxacin** |  | | | | | | | | |  |  |
| Total (234) | 0 | 0 | 0 | 3 | 52 | 179 | 0 | 0 | 0 | 27.107, <0.001 |  |
| *B. melitensis* (210) | 0 | 0 | 0 | 0 | 39 | 171 | 0 | 0 | 0 |  |  |
| *B. suis* (15) | 0 | 0 | 0 | 2 | 10 | 3 | 0 | 0 | 0 |  |  |
| *B. abortus* (5) | 0 | 0 | 0 | 1 | 2 | 2 | 0 | 0 | 0 |  |  |
| *B. amazoniensis* (4) | 0 | 0 | 0 | 0 | 1 | 3 | 0 | 0 | 0 |  |  |
| **Levofloxacin** |  | | | | | | | | |  |  |
| Total (234) | 0 | 0 | 0 | 3 | 54 | 156 | 21 | 0 | 0 | 29.504, <0.001 |  |
| *B. melitensis* (210) | 0 | 0 | 0 | 1 | 41 | 147 | 21 | 0 | 0 |  |  |
| *B. suis* (15) | 0 | 0 | 0 | 2 | 10 | 3 | 0 | 0 | 0 |  |  |
| *B. abortus* (5) | 0 | 0 | 0 | 0 | 3 | 2 | 0 | 0 | 0 |  |  |
| *B. amazoniensis* (4) | 0 | 0 | 0 | 0 | 0 | 4 | 0 | 0 | 0 |  |  |
| **Gentamicin** |  | | | | | | | | |  |  |
| Total (234) | 0 | 0 | 1 | 12 | 137 | 84 | 0 | 0 | 0 | 11.503, 0.0092 |  |
| *B. melitensis* (210) | 0 | 0 | 0 | 8 | 128 | 74 | 0 | 0 | 0 |  |  |
| *B. suis* (15) | 0 | 0 | 1 | 1 | 5 | 8 | 0 | 0 | 0 |  |  |
| *B. abortus* (5) | 0 | 0 | 0 | 3 | 2 | 0 | 0 | 0 | 0 |  |  |
| *B. amazoniensis* (4) | 0 | 0 | 0 | 0 | 2 | 2 | 0 | 0 | 0 |  |  |
| **Rifampicin** |  | | | | | | | | |  |  |
| Total (234) | 0 | 0 | 0 | 0 | 7 | 26 | 85 | 116 | 0 | 32.263, <0.001 |  |
| *B. melitensis* (210) | 0 | 0 | 0 | 0 | 1 | 18 | 78 | 113 | 0 |  |  |
| *B. suis* (15) | 0 | 0 | 0 | 0 | 4 | 6 | 4 | 1 | 0 |  |  |
| *B. abortus* (5) | 0 | 0 | 0 | 0 | 1 | 2 | 1 | 1 | 0 |  |  |
| *B. amazoniensis* (4) | 0 | 0 | 0 | 0 | 1 | 0 | 2 | 1 | 0 |  |  |
| **Tetracycline** |  | | | | | | | | |  |  |
| Total (234) | 0 | 21 | 58 | 135 | 20 | 0 | 0 | 0 | 0 | 41.248, <0.001 |  |
| *B. melitensis* (210) | 0 | 8 | 50 | 132 | 20 | 0 | 0 | 0 | 0 |  |  |
| *B. suis* (15) | 0 | 10 | 4 | 1 | 0 | 0 | 0 | 0 | 0 |  |  |
| *B. abortus* (5) | 0 | 1 | 2 | 2 | 0 | 0 | 0 | 0 | 0 |  |  |
| *B. amazoniensis* (4) | 0 | 2 | 2 | 0 | 0 | 0 | 0 | 0 | 0 |  |  |
| **Doxycycline** |  | | | | | | | | |  |  |
| Total (234) | 0 | 17 | 57 | 140 | 20 | 0 | 0 | 0 | 0 | 31.284, <0.001 |  |
| *B. melitensis* (210) | 0 | 9 | 50 | 134 | 17 | 0 | 0 | 0 | 0 |  |  |
| *B. suis* (15) | 0 | 8 | 5 | 1 | 1 | 0 | 0 | 0 | 0 |  |  |
| *B. abortus* (5) | 0 | 0 | 1 | 2 | 2 | 0 | 0 | 0 | 0 |  |  |
| *B. amazoniensis* (4) | 0 | 0 | 1 | 3 | 0 | 0 | 0 | 0 | 0 |  |  |
| **Trimethoprim-sulfamethoxazole** | | | | | | | | | |  |  |
| Total (234) | 20 | 127 | 51 | 36 | 0 | 0 | 0 | 0 | 0 | 2.730, 0.435 |  |
| *B. melitensis* (210) | 19 | 117 | 42 | 32 | 0 | 0 | 0 | 0 | 0 |  |  |
| *B. suis* (15) | 1 | 6 | 5 | 3 | 0 | 0 | 0 | 0 | 0 |  |  |
| *B. abortus* (5) | 0 | 2 | 2 | 1 | 0 | 0 | 0 | 0 | 0 |  |  |
| *B. amazoniensis* (4) | 0 | 2 | 2 | 0 | 0 | 0 | 0 | 0 | 0 |  |  |

## ^a^ Kruskal-Wallis H test and p-value

## In grey, the ECOFF breakpoints defined by EUCAST for each antibiotic.
