# Supplementary material for: Validation of EUCAST 2026 breakpoints for Brucella species using routine laboratory methods: A comprehensive French National Reference Centre study
Source: PLoS Negl Trop Dis. 2026 Oct 27;20(10):e0014817. doi: 10.1371/journal.pntd.0014817 (PMC13712602; doi:10.1371/journal.pntd.0014817)
Supplement: Supplementary file 3 [file pntd.0014817.s004.docx]

**S3 Table. MIC distribution for each antibiotic for Quality control values established on *Staphylococcus aureus* ATCC 29213 and *Streptococcus pneumoniae* ATCC 49619.**

|  |  | *Streptococcus pneumoniae* ATCC 49619 | | | *Staphylococcus aureus* ATCC 29213 | |
| --- | --- | --- | --- | --- | --- | --- |
| Antibiotic | **Method** | | **MIC Range (mg/L)** | **Zone Diameter (mm)** | **MIC Range (mg/L)** | **Zone Diameter (mm)** |
| Ceftriaxone | E-test | | 0.5-1.0 | ND | 0.5-1.0 | ND |
| Ciprofloxacin | E-test | | 0.5-1.0 | ND | 0.5-1.0 | ND |
| Gentamicin | E-test | | 0.5-1.0 | ND | 0.5-1.0 | ND |
| Levofloxacin | E-test | | 0.5-2.0 | ND | 1.0-2.0 | ND |
| Rifampicin | E-test | | 0.125-0.25 | ND | 0.06-0.125 | ND |
| Tetracycline | E-test | | 1.0-2.0 | ND | 0.25-1.0 | ND |
| Trimethoprim-sulfamethoxazole | E-test | | 0.25-0.5 | ND | 0.5-1.0 | ND |
| Doxycycline | E-test | | 1.0-2.0 | ND | 0.5-1.0 | ND |
| Streptomycin | Disk (10 μg) | | ND | 22-25 | ND | 24-27 |

ND, not determine
