# Supplementary material for: Validation of EUCAST 2026 breakpoints for Brucella species using routine laboratory methods: A comprehensive French National Reference Centre study
Source: PLoS Negl Trop Dis. 2026 Oct 27;20(10):e0014817. doi: 10.1371/journal.pntd.0014817 (PMC13712602; doi:10.1371/journal.pntd.0014817)
Supplement: Supplementary file 4 [file pntd.0014817.s005.docx]

This is a well-conducted study that addresses a critically important gap in clinical microbiology: the validation of newly established EUCAST breakpoints for *Brucella* species. The study leverages an impressive collection of 234 isolates from the French National Reference Centre, providing robust data that will directly impact clinical laboratory practice. The finding regarding streptomycin E-test methodology is particularly valuable and practice-changing.

- Abstract: "49.6% of isolates demonstrated rifampicin MICs at the EUCAST breakpoint"

This is slightly imprecise; they were within two dilutions of the ECOFF, not all at the breakpoint. Consider rephrasing to "within two dilutions of the breakpoint."

- Lines 87-89: When discussing emerging resistance, consider briefly mentioning the clinical significance - e.g., how rifampicin resistance (even reduced susceptibility) might affect relapse rates.
- Line 131: "incubation was performed at 35±2°C in 5% CO_2_ for 48 hours" - EUCAST recommends 35±1°C. While 2°C is the EUCAST allowable range, please clarify if this was a choice or a limitation.
- Line 149: Levene's test was used for homogeneity of variances, but with non-normal distributions, this may not be appropriate. Consider mentioning this as a limitation or using a non-parametric alternative.
- "E-test determination of streptomycin MICs ... yielded inconsistent results" - This is a critical finding, but the description is vague.
- Table 1: The abbreviation for “trimethoprim-sulfamethoxazole” should be “TMP-SMX”.
- The conclusion repeats much of the abstract. Consider revising and improving it.
- Check that all references are formatted consistently (some have DOIs, others do not).

The minor issues identified above should be addressed, particularly clarifying the streptomycin E-test inconsistency and ensuring consistency in data presentation. With these revisions, the manuscript will be a valuable on brucellosis diagnostics and antimicrobial susceptibility testing.
