# Supplementary material for: Validation of EUCAST 2026 breakpoints for Brucella species using routine laboratory methods: A comprehensive French National Reference Centre study
Source: PLoS Negl Trop Dis. 2026 Oct 27;20(10):e0014817. doi: 10.1371/journal.pntd.0014817 (PMC13712602; doi:10.1371/journal.pntd.0014817)
Supplement: Supplementary file 5 [file pntd.0014817.s007.docx]

**Response to the Academic Editor and Journal Requirements**

**Editor comment**

Your manuscript has been reviewed by 3 experts, each of who considered it a valuable contribution, but each also had comments and suggestions for improvement.

**Response**: We thank the Editor. Every point raised by the three reviewers has been addressed individually below, and all corresponding modifications are visible in the marked-up manuscript.

**Journal Requirement 1.** In the online submission form you indicate that your data is not available for proprietary reasons and have provided a contact point for accessing this data… the contact point must not be an author on the manuscript and must be an institutional contact, ideally not an individual.

**Response**: We apologize for this oversight. The data access contact has been changed from an author to a non-author institutional body. The Data Availability Statement has been updated within the submission form to read:

All data underlying the findings are held by the French National Reference Centre for Brucellosis, Nîmes University Hospital. The isolates and associated clinical metadata derive from a mandatory national surveillance activity and contain potentially identifying patient information, so they cannot be placed in a public repository under French data protection law (CNIL). De-identified data are available to qualified researchers on reasonable request, subject to approval by the institutional body responsible for research data governance, which is not composed of authors of this study:

Direction de la Recherche Clinique et de l’Innovation (DRCI), Centre Hospitalier Universitaire de Nîmes, Place du Professeur Robert Debré, 30029 Nîmes Cedex 9, France. E-mail: drc@chu-nimes.fr. Telephone: +33 (0)4 66 68 42 36.

Requests are reviewed by the DRCI together with the Institutional Review Board of Nîmes University Hospital (approval 2024.03.05). Our institution has additionally appointed an Open Science Officer Director (Dr Chris Serrand), who assesses the scientific merit of each request.

**Journal Requirement 2**. We have amended your Competing Interest statement to comply with journal style. We kindly ask that you double check the statement and let us know if anything is incorrect.

**Response:** We have checked the amended statement and confirm it is correct. The authors have no competing interests to declare.

**Journal Requirement 3 - ORCID**:

When completing the submission form, we were unable to enter the corresponding author’s ORCID identifier in the system. The identifiers of the authors who hold one are listed below, and we would be grateful for the editorial office’s assistance in attaching them to the submission record:

Albert Sotto - 0000-0002-6463-9386

David O'Callaghan - 0000-0003-4350-6143

Anne Keriel - 0000-0003-3340-299X

Jean-Philippe Lavigne - 0000-0002-9484-0304

# **Reviewer(s)’ Comments to Author**

**Reviewer 1**

We thank Reviewer 1 for a very thorough and encouraging assessment, and in particular for the observation that brucellosis requires greater political and professional engagement. The comments have improved both the accessibility and the scope of the paper.

**Comment 1.1.** The methods used to develop the study were appropriately described and are aligned with the proposed objectives.

**Response.** We thank the reviewer.

**Comment 1.2.** To facilitate the visualization of the methodological steps performed in this study, I suggest including a flowchart summarizing this information.

**Response.** We thank the reviewer for this constructive suggestion. We have created a new Fig 1, a flow diagram covering the study workflow from isolate reception and the inclusion and exclusion criteria, through hierarchical species identification and the species breakdown, to antimicrobial susceptibility testing and interpretation against EUCAST 2026 criteria. The former Fig 1 has been renumbered Fig 2, and the in-text citation and figure legend have been updated accordingly.

**Comment 1.3.** Statistical analyzes performed are suitable for the context of the study.

**Response.** We thank the reviewer. We note that Reviewer 3 identified one specific point, the use of Levene’s test with non-normal data, which has been corrected as described under Comment 3.4.

**Comment 1.4.** The results of the manuscript are plausible.

**Response.** We thank the reviewer.

**Comment 1.5.** The study describes that 234 Brucella isolates were analyzed; however, the legend in Table S1 indicates that 215 Brucella isolates were analyzed. This information needs to be reviewed to ensure it is aligned.

**Response.** We thank the reviewer for catching it. The typographical error is now corrected. The legend of S1 Table was updated.

**Comment 1.6.** Lines 220-222 state, “Seven of eight antibiotics (ceftriaxone, ciprofloxacin, levofloxacin, rifampicin, doxycycline, and tetracycline) showed statistically significant differences in MIC distributions among species,” however, six antibiotics were listed in parentheses. Review this information.

**Response.** The reviewer is correct, and we thank them for this careful reading. Gentamicin had been inadvertently omitted from the parenthetical list. Gentamicin did show a statistically significant difference between species (Kruskal-Wallis H = 11.503, p = 0.0093), so the count of seven was correct and the list has been completed.

**Comment 1.7.** The findings are relevant to the objectives of the study. The tables and figures are clear and legible.

**Response.** We thank the reviewer.

**Comment 1.8.** The conclusions are supported by the data presented. The limitations of the study were clearly addressed and discussed in the manuscript. The discussion about the relevance of the study to public health needs to be broadened.

**Response.** We agree that the public health dimension was underdeveloped, and we thank the reviewer for pressing us on it. A new paragraph has been added to the Discussion, developing three points: that because 65.4% of our isolates were acquired in Africa and 12.4% in the Middle East, this collection is in practice a window onto the populations circulating in endemic regions where systematic AST is rarely feasible; that the streptomycin finding has its greatest operational impact in exactly those settings, where gradient strips are preferred for their simplicity and where an uncorrected artefact would generate spurious reports of aminoglycoside resistance and distort regional estimates; and that the demonstration that method choice alone can move an apparent resistance rate from 0% to 8% argues for treating AST harmonization as a surveillance priority in its own right.

**Comment 1.9.** Some paragraphs in the manuscript were written in a very long way, making reading tiring. I suggest that long paragraphs be divided.

**Response.** We modified in the Methods (antimicrobial susceptibility testing), the Results (species-specific susceptibility profiles and streptomycin testing) and the Discussion (the streptomycin finding).

**Comment 1.10.** I strongly recommend that the “Introduction” section briefly describe the importance of using the “One Health” approach to combat neglected zoonotic diseases, such as brucellosis. Below is a suggested article… Meurer IR. Infect Drug Resist. 2025;18:5007-5008.

**Response.** We thank the reviewer warmly for this suggestion, which we consider an important addition. Meurer article has been cited as new reference [15] and a new paragraph has been added to the Introduction.

**Comment 1.11.** Briefly describe the prospects for future research based on the results found in this study.

**Response.** We thank the reviewer for this suggestion. A new paragraph has been added at the end of the Discussion.

**Comment 1.12.** The title is well written and reflects the focus of the manuscript well. The abstract presents the main information described in the manuscript.

**Response.** We thank the reviewer. The title is unchanged. One sentence of the Abstract has been made more precise following Comment 3.1.

**Reviewer 2**:

**Comment 2.1.** The MIC breakpoints provided in the EUCAST Clinical Breakpoint Tables v. 16.1 are based on the broth microdilution method. Is it acceptable to use E-test-derived MICs to interpret susceptibility against these EUCAST breakpoints? The authors should justify the methodological equivalence or discuss the limitations of using E-test in this context.

**Response.** This is a fair and important challenge, and we are grateful for it. The reviewer is entirely correct that EUCAST breakpoints and ECOFFs are formally derived from ISO 20776-1 broth microdilution. We should have addressed this explicitly rather than leaving it implicit.

The Methods now explain why gradient diffusion was chosen. Broth microdilution is poorly suited to routine work with Brucella, a slow-growing, fastidious Hazard Group 3 organism for which the preparation, inoculation and reading of microdilution panels both increase technical variability and substantially increase laboratory biorisk; manipulating this organism in liquid medium in microdilution format carries a well-recognized aerosol risk, and brucellosis remains one of the most frequently reported laboratory-acquired infections. The choice of a solid-medium method is therefore, for Brucella, partly a biosafety decision, and it is also consistent with the stated aim of the study, which was to validate the breakpoints under the conditions actually used by clinical laboratories

We have added the direct comparative study of Gür et al. (Antimicrob Agents Chemother 1999;43:2337) as new reference [19]. These authors compared E-test with broth microdilution for *B. melitensis*, and their data are unusually pertinent here: agreement within fourfold dilutions was 100% for doxycycline, 95% for azithromycin and 91% for rifampin, but only 82% for streptomycin, the lowest of all agents tested together with ofloxacin. This literature converges with our own observation, and the Discussion now says so explicitly, together with a proposed physicochemical explanation based on the poor diffusion of a highly polar aminoglycoside through MH-F agar supplemented with horse blood and β-NAD.

Finally, a corresponding limitation has been added to the Discussion, acknowledging that method-dependent shifts in MIC of one doubling dilution cannot be excluded for individual agents and that the streptomycin data show such shifts may occasionally be larger.

**Comment 2.2.** I would suggest that the authors include in the main text the treatment details for the patients from whom the isolates were obtained, including the types and durations of antibiotics used. This would allow an assessment of whether the in vitro susceptibility results correlate with clinical outcomes, which would greatly strengthen the clinical relevance of the study.

**Response.** We agree that such an analysis would materially strengthen the clinical relevance of the work, and we thank the reviewer for the suggestion. Unfortunately, we are not able to provide it for this collection. As a National Reference Centre, we receive isolates accompanied by a limited epidemiological questionnaire; detailed therapeutic information (the agents actually prescribed, their doses and the duration of treatment) is not systematically transmitted with the strains, and patient-level outcome data are not available to us for the great majority of the 234 isolates. Presenting a correlation based on the incomplete subset that we do hold would, in our view, risk being misleading.

What we can state is that, in France, the combination of doxycycline and rifampicin is the regimen of choice in the large majority of cases (>90%), with an aminoglycoside or another antibiotic class used when the initial regimen is not tolerated or when focal disease is present, and that no relapse was reported to the Centre in a patient infected with an isolate whose rifampicin MIC lay within two doubling dilutions of the ECOFF. We would emphasize that isolates from relapsing patients are systematically referred to the National Reference Centre, so that any emergence of reduced rifampicin susceptibility following treatment would have been detected by this surveillance. The absence of such a signal is therefore informative rather than simply reflecting missing data.

We have added this explicitly to the limitations of the study.

**Comment 2.3.** This study analysed the antimicrobial susceptibility of 234 Brucella isolates from France, enriching the surveillance data on Brucella resistance and providing useful information for the clinical selection of therapeutic agents for brucellosis.

**Response.** We thank the reviewer for this positive summary.

**Reviewer 3** :

**Comment 3.1.** Abstract: “49.6% of isolates demonstrated rifampicin MICs at the EUCAST breakpoint”. This is slightly imprecise; they were within two dilutions of the ECOFF, not all at the breakpoint. Consider rephrasing to “within two dilutions of the breakpoint.”

**Response.** We modified accordingly.

**Comment 3.2.** Lines 87-89: When discussing emerging resistance, consider briefly mentioning the clinical significance - e.g., how rifampicin resistance (even reduced susceptibility) might affect relapse rates.

**Response.** We agree and thank the reviewer. A sentence has been added to the Introduction.

**Comment 3.3.** Line 131: “incubation was performed at 35±2°C in 5% CO2 for 48 hours” - EUCAST recommends 35±1°C. While 2°C is the EUCAST allowable range, please clarify if this was a choice or a limitation.

**Response.** We thank the reviewer for this careful check. This was neither a deliberate choice nor a limitation but an error of transcription in the manuscript. We corrected accordingly in the new version.

**Comment 3.4.** Line 149: Levene’s test was used for homogeneity of variances, but with non-normal distributions, this may not be appropriate. Consider mentioning this as a limitation or using a non-parametric alternative.

**Response.** We thank the reviewer for the observation. Levene’s test is itself sensitive to departures from normality, so applying it to data already shown to be non-normal was not appropriate. Because the subsequent analysis is entirely rank-based and does not require homogeneity of variances, we have removed the test rather than substituting another, and we have also deleted a later sentence that merely restated the Shapiro-Wilk rationale. We modified the Method section. No numerical result is affected, since Levene’s test had not been used to select the subsequent analyses; these had been chosen on the basis of the Shapiro-Wilk results.

**Comment 3.5.** *“*E-test determination of streptomycin MICs … yielded inconsistent results” - This is a critical finding, but the description is vague.

**Response.** We agree that the original description was too vague for what is the central methodological claim of the paper, and we thank the reviewer for insisting on precision. The passage in the Results has been rewritten to specify what was discordant, with what, and in what direction: the E-test results were discordant both with the disk diffusion data obtained on the same isolates and with the wild-type distribution expected for this genus; among the first 150 isolates tested, 12 (8.0%) had streptomycin MICs above the EUCAST ECOFF and would therefore have been reported as non-wild-type, while the distribution as a whole was displaced upwards relative to that obtained by disk diffusion. Strips from a second manufacturer (i2a), of a different production lot, were then evaluated on the same 150 isolates and reproduced the elevated MICs, excluding a lot- or manufacturer-specific defect. The Discussion now offers both a literature precedent and a physicochemical explanation for the artefact.

**Comment 3.6.** Table 1: The abbreviation for “trimethoprim-sulfamethoxazole” should be “TMP-SMX”.

**Response.** Corrected. The abbreviation has been changed from SXT to TMP-SMX both in the body of Table 1 and in the table footnote, and TMP-SMX is also used in the new Fig 1.

**Comment 3.7.** The conclusion repeats much of the abstract. Consider revising and improving it.

**Response.** We have been rewritten this conclusion.

**Comment 3.8.** Check that all references are formatted consistently (some have DOIs, others do not).

**Response.** The reference list has been harmonized to PLOS (Vancouver) style throughout. DOIs have been added to all references for which one exists.

**Comment 3.9.** The minor issues identified above should be addressed, particularly clarifying the streptomycin E-test inconsistency and ensuring consistency in data presentation. With these revisions, the manuscript will be a valuable [contribution] on brucellosis diagnostics and antimicrobial susceptibility testing.

**Response.** We thank the reviewer for this generous assessment. The streptomycin passage has been substantially expanded (Comments 2.1 and 3.5) and every numerical value in the manuscript, tables, figures and supporting information has been re-verified against the final 234-isolate dataset.
