# Supplementary material for: Validation of EUCAST 2026 breakpoints for Brucella species using routine laboratory methods: A comprehensive French National Reference Centre study
Source: PLoS Negl Trop Dis. 2026 Oct 27;20(10):e0014817. doi: 10.1371/journal.pntd.0014817 (PMC13712602; doi:10.1371/journal.pntd.0014817)
Supplement: Peer review history

# Peer review: Decision Letter 0

**DOI:** 10.1371/journal.pntd.0014817.r001

**Authors:** Richard Bowen, Stuart Blacksell

12 Aug 2026

PNTD-D-26-01374

Validation of EUCAST 2026 breakpoints for Brucella species using routine laboratory methods: A comprehensive French National Reference Centre study

PLOS Neglected Tropical Diseases

Dear Dr. Lavigne,

Thank you for submitting your manuscript to PLOS Neglected Tropical Diseases. After careful consideration, we feel that it has merit but does not fully meet PLOS Neglected Tropical Diseases's publication criteria as it currently stands. Therefore, we invite you to submit a revised version of the manuscript that addresses the points raised during the review process.

Please submit your revised manuscript within by Oct 11 2026 11:59PM. If you will need more time than this to complete your revisions, please reply to this message or contact the journal office at plosntds@plos.org. When you're ready to submit your revision, log on to https://www.editorialmanager.com/pntd/ and select the 'Submissions Needing Revision' folder to locate your manuscript file.

Please include the following items when submitting your revised manuscript:

* A letter that responds to each point raised by the editor and reviewer(s). You should upload this letter as a separate file labeled 'Response to Reviewers'. This file does not need to include responses to any formatting updates and technical items listed in the 'Journal Requirements' section below.

* A marked-up copy of your manuscript that highlights changes made to the original version. You should upload this as a separate file labeled 'Revised Manuscript with Track Changes'.

* An unmarked version of your revised paper without tracked changes. You should upload this as a separate file labeled 'Manuscript'.

If you would like to make changes to your financial disclosure, competing interests statement, or data availability statement, please make these updates within the submission form at the time of resubmission. Guidelines for resubmitting your figure files are available below the reviewer comments at the end of this letter.

As the corresponding author, your ORCID iD is verified in the submission system and will appear in the published article. PLOS supports the use of ORCID, and we encourage all coauthors to register for an ORCID iD and use it as well. Please encourage your coauthors to verify their ORCID iD within the submission system before final acceptance, as unverified ORCID iDs will not appear in the published article. Only the individual author can complete the verification step; PLOS staff cannot verify ORCID iDs on behalf of authors.

We look forward to receiving your revised manuscript.

Kind regards,

Richard A. Bowen, DVM PhD

Academic Editor

PLOS Neglected Tropical Diseases

Stuart Blacksell

Section Editor

PLOS Neglected Tropical Diseases

Shaden Kamhawi

co-Editor-in-Chief

PLOS Neglected Tropical Diseases

orcid.org/0000-0003-4304-636XX

Paul Brindley

co-Editor-in-Chief

PLOS Neglected Tropical Diseases

orcid.org/0000-0003-1765-0002

**Additional Editor Comments (if provided):**

Your manuscript has been reviewed by 3 experts, each of who considered it a valuable contribution, but each also had comments and suggestions for improvement. Please evaluate reviewer comments carefully, edit your manuscript as you feel best, then resubmit along with a document providing a point-by-point response to reviewer comments. Thank you.

**Journal Requirements:**

If the reviewer comments include a recommendation to cite specific previously published works, please review and evaluate these publications to determine whether they are relevant and should be cited. There is no requirement to cite these works unless the editor has indicated otherwise.

1) In the online submission form you indicate that your data is not available for proprietary reasons and have provided a contact point for accessing this data. Please note that your current contact point is a co-author on this manuscript. According to our Data Policy, the contact point must not be an author on the manuscript and must be an institutional contact, ideally not an individual. Please revise your data statement to a non-author institutional point of contact, such as a data access or ethics committee, and send this to us via return email. Please also include contact information for the third party organization, and please include the full citation of where the data can be found.

2) We have amended your Competing Interest statement to comply with journal style. We kindly ask that you double check the statement and let us know if anything is incorrect.

**Reviewers' Comments:**

Reviewer's Responses to Questions

**Key Review Criteria Required for Acceptance?**

As you describe the new analyses required for acceptance, please consider the following:

**Methods**

-Are the objectives of the study clearly articulated with a clear testable hypothesis stated?

-Is the study design appropriate to address the stated objectives?

-Is the population clearly described and appropriate for the hypothesis being tested?

-Is the sample size sufficient to ensure adequate power to address the hypothesis being tested?

-Were correct statistical analysis used to support conclusions?

-Are there concerns about ethical or regulatory requirements being met?

Reviewer #1: - The methods used to develop the study were appropriately described and are aligned with the proposed objectives.

- To facilitate the visualization of the methodological steps performed in this study, I suggest including a flowchart summarizing this information.

- Statistical analyzes performed are suitable for the context of the study.

Reviewer #2: The MIC breakpoints provided in the EUCAST Clinical Breakpoint Tables v. 16.1 are based on the broth microdilution method. Is it acceptable to use E‑test‐derived MICs to interpret susceptibility against these EUCAST breakpoints? The authors should justify the methodological equivalence or discuss the limitations of using E‑test in this context.

Reviewer #3: (No Response)

**********

**Results**

-Does the analysis presented match the analysis plan?

-Are the results clearly and completely presented?

-Are the figures (Tables, Images) of sufficient quality for clarity?

Reviewer #1: - The results of the manuscript are plausible.

- The study describes that 234 Brucella isolates were analyzed; however, the legend in Table S1 indicates that 215 Brucella isolates were analyzed. This information needs to be reviewed to ensure it is aligned.

- Lines 220-222 state, “Seven of eight antibiotics (ceftriaxone, ciprofloxacin, levofloxacin, rifampicin, doxycycline, and tetracycline) showed statistically significant differences in MIC distributions among species,” however, six antibiotics were listed in parentheses. Review this information.

- The findings are relevant to the objectives of the study.

- The tables and figures are clear and legible.

Reviewer #2: I would suggest that the authors include in the main text the treatment details for the patients from whom the isolates were obtained, including the types and durations of antibiotics used. This would allow an assessment of whether the in vitro susceptibility results correlate with clinical outcomes, which would greatly strengthen the clinical relevance of the study.

Reviewer #3: (No Response)

**********

**Conclusions**

-Are the conclusions supported by the data presented?

-Are the limitations of analysis clearly described?

-Do the authors discuss how these data can be helpful to advance our understanding of the topic under study?

-Is public health relevance addressed?

Reviewer #1: - The conclusions are supported by the data presented.

- The limitations of the study were clearly addressed and discussed in the manuscript.

- The discussion about the relevance of the study to public health needs to be broadened.

Reviewer #2: YES

Reviewer #3: (No Response)

**********

**Editorial and Data Presentation Modifications?**

Use this section for editorial suggestions as well as relatively minor modifications of existing data that would enhance clarity. If the only modifications needed are minor and/or editorial, you may wish to recommend “Minor Revision” or “Accept”.

Reviewer #1: (No Response)

Reviewer #2: (No Response)

Reviewer #3: (No Response)

**********

**Summary and General Comments**

Use this section to provide overall comments, discuss strengths/weaknesses of the study, novelty, significance, general execution and scholarship. You may also include additional comments for the author, including concerns about dual publication, research ethics, or publication ethics. If requesting major revision, please articulate the new experiments that are needed.

Reviewer #1: Dear authors,

Brucellosis is one of several neglected zoonotic diseases that require greater political and professional engagement to mitigate their impacts. This study is relevant to public health in the context of combating antimicrobial resistance, acting in the validation of EUCAST 2026 breakpoints for Brucella species. The results obtained provide important practical guidance for clinical microbiology laboratories, and also offer insights for the development of future research in this field. The manuscript is well structured, however, some points need to be presented, adjusted, and discussed.

- The title is well written and reflects the focus of the manuscript well.

- The abstract presents the main information described in the manuscript.

- Some paragraphs in the manuscript were written in a very long way, making reading tiring. I suggest that long paragraphs be divided.

- I strongly recommend that the “Introduction” section briefly describe the importance of using the "One Health" approach to combat neglected zoonotic diseases, such as brucellosis. Below is a suggested article for writing this information in general terms (see the fourth and fifth paragraphs of this article):

*Meurer IR. The Importance of Medical Knowledge About Q Fever in the Context of Timely Diagnosis and Treatment and the Use of the One Health Approach in Combating This and Other Neglected Zoonotic Diseases [Letter]. Infect Drug Resist. 2025;18:5007-5008. https://doi.org/10.2147/IDR.S567142

- Briefly describe the prospects for future research based on the results found in this study.

Reviewer #2: This study analysed the antimicrobial susceptibility of 234 Brucella isolates from France, enriching the surveillance data on Brucella resistance and providing useful information for the clinical selection of therapeutic agents for brucellosis.

Reviewer #3: (No Response)

**********

PLOS authors have the option to publish the peer review history of their article (what does this mean?). If published, this will include your full peer review and any attached files.

If you choose “no”, your identity will remain anonymous but your review may still be made public.

**Do you want your identity to be public for this peer review?**  For information about this choice, including consent withdrawal, please see our Privacy Policy.

Reviewer #1: No

Reviewer #2: **Yes:**  Hai Jiang

Reviewer #3: **Yes:**  Chaowen Deng

[NOTE: If reviewer comments were submitted as an attachment file, they will be attached to this email and accessible via the submission site. Please log into your account, locate the manuscript record, and check for the action link "View Attachments". If this link does not appear, there are no attachment files.]

**Figure resubmission:**

**Reproducibility:**

To enhance the reproducibility of your results, we recommend that authors of applicable studies deposit laboratory protocols in protocols.io, where a protocol can be assigned its own identifier (DOI) such that it can be cited independently in the future. Additionally, PLOS ONE offers an option to publish peer-reviewed clinical study protocols. Read more information on sharing protocols at https://plos.org/protocols?utm_medium=editorial-email&utm_source=authorletters&utm_campaign=protocols

Attachment Submitted filename: Comments for PNTD-D-26-01374.docx

# Author comment: Author response to Decision Letter 1

**DOI:** 10.1371/journal.pntd.0014817.r002

17 Sep 2026

Attachment Submitted filename: Response to the Reviewers.docx

# Peer review: Decision Letter 1

**DOI:** 10.1371/journal.pntd.0014817.r003

**Authors:** Richard Bowen, Stuart Blacksell

4 Oct 2026

Dear Professeur Lavigne,

We are pleased to inform you that your manuscript 'Validation of EUCAST 2026 breakpoints for Brucella species using routine laboratory methods: A comprehensive French National Reference Centre study' has been provisionally accepted for publication in PLOS Neglected Tropical Diseases.

Before your manuscript can be formally accepted you will need to complete some formatting changes, which you will receive in a follow up email. A member of our team will be in touch with a set of requests.

Please note that your manuscript will not be scheduled for publication until you have made the required changes, so a swift response is appreciated.

IMPORTANT: The editorial review process is now complete. PLOS will only permit corrections to spelling, formatting or significant scientific errors from this point onwards. Requests for major changes, or any which affect the scientific understanding of your work, will cause delays to the publication date of your manuscript.

Should you, your institution's press office or the journal office choose to press release your paper, you will automatically be opted out of early publication. We ask that you notify us now if you or your institution is planning to press release the article. All press must be co-ordinated with PLOS.

Thank you again for supporting Open Access publishing; we are looking forward to publishing your work in PLOS Neglected Tropical Diseases.

Best regards,

Richard A. Bowen, DVM PhD

Academic Editor

PLOS Neglected Tropical Diseases

Stuart Blacksell

Section Editor

PLOS Neglected Tropical Diseases

Shaden Kamhawi

co-Editor-in-Chief

PLOS Neglected Tropical Diseases

orcid.org/0000-0003-4304-636XX

Paul Brindley

co-Editor-in-Chief

PLOS Neglected Tropical Diseases

orcid.org/0000-0003-1765-0002

***********************************************************

Thank you for the comprehensive editing and revision of your manuscript. The reviewers find this valuable work now acceptable for publication.

Reviewer's Responses to Questions

**Key Review Criteria Required for Acceptance?**

As you describe the new analyses required for acceptance, please consider the following:

**Methods**

-Are the objectives of the study clearly articulated with a clear testable hypothesis stated?

-Is the study design appropriate to address the stated objectives?

-Is the population clearly described and appropriate for the hypothesis being tested?

-Is the sample size sufficient to ensure adequate power to address the hypothesis being tested?

-Were correct statistical analysis used to support conclusions?

-Are there concerns about ethical or regulatory requirements being met?

Reviewer #1: The changes made improved the quality and scientific relevance of the manuscript.

Reviewer #2: the newly added text in lines 159–168 concerning the rationale for the choice of experimental method is appreciated. However, this content would be more appropriate in the Discussion section, specifically within the limitations analysis. Please move this passage to the Discussion and integrate it with the existing discussion of methodological limitations. It should also be clearly stated that E‑test is not the reference method for EUCAST breakpoints and that this represents a limitation of the study.

Reviewer #3: The study clearly articulated with a clear testable hypothesis stated

**********

**Results**

-Does the analysis presented match the analysis plan?

-Are the results clearly and completely presented?

-Are the figures (Tables, Images) of sufficient quality for clarity?

Reviewer #1: The changes made improved the quality and scientific relevance of the manuscript.

Reviewer #2: the treatment details of the patients—including the types of antibiotics administered and the duration of therapy—have not been supplemented or explained in the revised manuscript. In addition, the Methods section (lines 124–125) states that clinical presentation and documentation of prior relapse were collected; however, these data are not presented in the Results section. Please provide these data, or, if they are unavailable, explicitly acknowledge this and discuss it as a limitation.

Reviewer #3: The analysis presented match the analysis plan.

**********

**Conclusions**

-Are the conclusions supported by the data presented?

-Are the limitations of analysis clearly described?

-Do the authors discuss how these data can be helpful to advance our understanding of the topic under study?

-Is public health relevance addressed?

Reviewer #1: The changes made improved the quality and scientific relevance of the manuscript.

Reviewer #2: (No Response)

Reviewer #3: The conclusions supported by the data presented.

**********

**Editorial and Data Presentation Modifications?**

Use this section for editorial suggestions as well as relatively minor modifications of existing data that would enhance clarity. If the only modifications needed are minor and/or editorial, you may wish to recommend “Minor Revision” or “Accept”.

Reviewer #1: (No Response)

Reviewer #2: (No Response)

Reviewer #3: Accept.

**********

**Summary and General Comments**

Use this section to provide overall comments, discuss strengths/weaknesses of the study, novelty, significance, general execution and scholarship. You may also include additional comments for the author, including concerns about dual publication, research ethics, or publication ethics. If requesting major revision, please articulate the new experiments that are needed.

Reviewer #1: Dear authors,

The changes made improved the quality and scientific relevance of the manuscript. Therefore, I recommend its publication.

Reviewer #2: (No Response)

Reviewer #3: No further comment.

**********

PLOS authors have the option to publish the peer review history of their article (what does this mean?). If published, this will include your full peer review and any attached files.

If you choose “no”, your identity will remain anonymous but your review may still be made public.

**Do you want your identity to be public for this peer review?**  For information about this choice, including consent withdrawal, please see our Privacy Policy.

Reviewer #1: No

Reviewer #2: No

Reviewer #3: **Yes:**  Dr. Chaowen Deng

# Editor report: Acceptance letter

**DOI:** 10.1371/journal.pntd.0014817.r004

**Authors:** Richard Bowen, Stuart Blacksell

Dear Professeur Lavigne,

We are delighted to inform you that your manuscript, "Validation of EUCAST 2026 breakpoints for Brucella species using routine laboratory methods: A comprehensive French National Reference Centre study," has been formally accepted for publication in PLOS Neglected Tropical Diseases.

We have now passed your article onto the PLOS Production Department who will complete the rest of the publication process. All authors will receive a confirmation email upon publication.

The corresponding author will soon be receiving a typeset proof for review, to ensure errors have not been introduced during production. Please review the PDF proof of your manuscript carefully, as this is the last chance to correct any scientific or type-setting errors. Please note that major changes, or those which affect the scientific understanding of the work, will likely cause delays to the publication date of your manuscript. Note: Proofs for Front Matter articles (Editorial, Viewpoint, Symposium, Review, etc...) are generated on a different schedule and may not be made available as quickly.

Soon after your final files are uploaded, the early version of your manuscript will be published online unless you opted out of this process. The date of the early version will be your article's publication date. The final article will be published to the same URL, and all versions of the paper will be accessible to readers.

For Research Articles, you will receive an invoice from PLOS for your publication fee after your manuscript has reached the completed accept phase. If you receive an email requesting payment before acceptance or for any other service, this may be a phishing scheme. Learn how to identify phishing emails and protect your accounts at https://explore.plos.org/phishing.

Thank you again for supporting open-access publishing; we are looking forward to publishing your work in PLOS Neglected Tropical Diseases.

Best regards,

Shaden Kamhawi

co-Editor-in-Chief

PLOS Neglected Tropical Diseases

Paul Brindley

co-Editor-in-Chief

PLOS Neglected Tropical Diseases
